# A universal null-distribution for topological data analysis

**DOI:** 10.1038/s41598-023-37842-2

**Published:** 2023-07-28

**Authors:** Omer Bobrowski, Primoz Skraba

**Affiliations:** 1 Viterbi Faculty of Electrical and Computer Engineering, Technion - Israel Institute of Technology, Haifa, Israel; 2 School of Mathematical Sciences, Queen Mary University of London, London, UK

**Keywords:** Applied mathematics, Computational science, Statistics

## Abstract

One of the most elusive challenges within the area of topological data analysis is understanding the distribution of persistence diagrams arising from data. Despite much effort and its many successful applications, this is largely an open problem. We present a surprising discovery: normalized properly, persistence diagrams arising from random point-clouds obey a universal probability law. Our statements are based on extensive experimentation on both simulated and real data, covering point-clouds with vastly different geometry, topology, and probability distributions. Our results also include an explicit well-known distribution as a candidate for the universal law. We demonstrate the power of these new discoveries by proposing a new hypothesis testing framework for computing significance values for individual topological features within persistence diagrams, providing a new quantitative way to assess the significance of structure in data.

## Introduction

Topological Data Analysis (TDA) focuses on extracting structural information from data, in order to enhance their processing in statistics and machine learning. This field has been rapidly developing over the past two decades, bringing together mathematicians, statisticians, computer scientists, engineers, and data scientists. The motivation behind TDA is that topological methods are highly versatile, coordinate-free, and robust to various deformations^1^. Topological methods have been applied successfully in numerous applications, in areas such as neuroscience^2–4^, medicine and biology^5–8^, material science^9,10^, dynamical systems^11,12^, and cosmology^13,14^.

One of the key challenges in TDA is to distinguish between “signal”—meaningful structures underlying the data, and “noise”—features that arise from the local randomness and inaccuracies within the data^15–17^. The most prominent solution developed in TDA to address this issue is *persistent homology*. Briefly, it identifies structures such as holes and cavities (“air pockets”) formed by the data, and records the scales at which they are created and terminated (*birth* and *death*, respectively). The common practice in TDA has been to use this birth-death information to assess the statistical significance of topological features^18–21^. However, research so far has yet to provide an approach which is generic, robust, and theoretically justified. A parallel line of research has been the theoretical probabilistic analysis of persistent homology generated by random data, as means to establish a null-distribution. While this direction has been fruitful^22–25^, its use in practice has been limited. The main gap between theory and practice is that these studies indicate that the distribution of noise in persistent homology: (a) does not have a simple closed-form description, and (b) strongly depends on the model generating the point-cloud.

Our main goal in this paper is to refute the last premise, and to make the case that the distribution of noise in persistent homology of random point-clouds is in fact *universal*. Specifically, we claim that the limiting distribution of *persistence values* (measured using the death/birth ratio) is independent of the model generating the point-cloud. This result is loosely analogous to the central limit theorem, where sums of many different types of random variables always converge to the normal distribution. The emergence of such universality for persistence diagrams is highly surprising.

We support our universality statements by an extensive body of experiments, including point-clouds generated by different geometries, topologies, and probability distributions. These include simulated data as well as data from real-world applications (image processing, signal processing, and natural language processing). Our main goal here is to introduce the unexpected behavior of statistical universality in persistence diagrams, in order to initiate a shift of paradigm in stochastic topology that will lead to the development of a new theory. Developing this new theory, and proving the conjectures made here, is anticipated to be an exciting yet a challenging long journey, and is outside the scope of this paper. Based on our universality conjectures, we develop a powerful hypothesis testing framework for persistence diagrams, allowing us to compute numerical significance measures for individual features using very few assumptions on the underlying model.

## Persistent homology for point-clouds

In order to approximate the structure formed by point-clouds, a common practice in TDA is to construct a special type of hypergraphs known as *geometric simplicial complexes*, whose faces are determined by the spatial configuration of the points. We consider the two most commonly used constructions— the **Čech** and the **Vietoris-Rips** complexes, both are parameterized by a scale parameter *r* (see “Methods” section). The *homology groups* ($\textrm{H}_k$) of a simplicial complex capture structural information about the complex. Loosely speaking, $\textrm{H}_0$ contains information about connected components, $\textrm{H}_1$—about closed loops surrounding holes, $\textrm{H}_2$—about closed surfaces surrounding cavities (“air pockets”). Generally, we say that $\textrm{H}_k$ describes ‘*k*-dimensional cycles’. For more details, see “Methods”.

**Figure 1:**
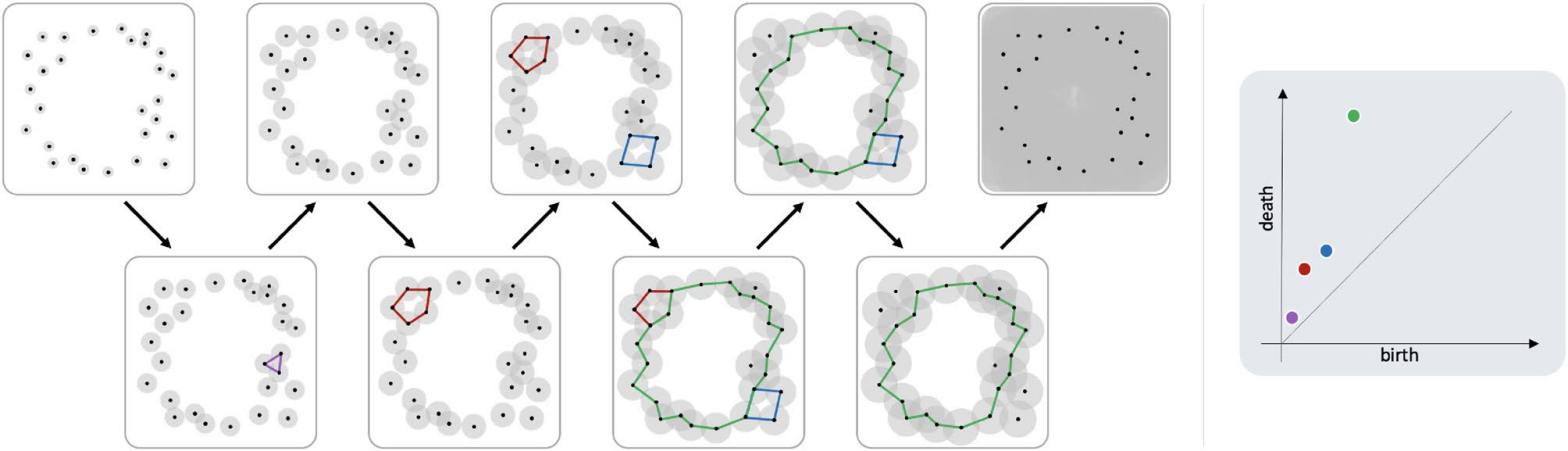
A persistence diagram generated by a point-cloud sampled from a noisy circle. The shape studied here is the union of balls (in gray), which is equivalent to the Čech complex. As the radius  *r* increases, four different 1-cycles appear (in different color). The radii at which each cycle appears and filled are recorded by the persistence diagram on the right. The green cycle stands out, being the furthest from the diagonal, and indeed this cycle is a only “real” feature of the sample, while the others can be considered as “noise”.

For both the Čech and Rips complexes, $\textrm{H}_k$ is highly sensitive to the choice of the scale parameter *r*. To overcome this, *persistent homology* considers the entire range of scales, tracking the evolution of *k*-cycles as the value of *r* increases. In this process (called a ‘filtration’), cycles are created (born) and later filled-in (die). This information is most often represented via *persistence diagrams*, 2-dimensional scatter plots, where each point $p=(\textrm{b},\textrm{d})\in \mathbb {R}^2$ represents the *birth* and *death* times (scales) of a single cycle in the filtration, see Fig. 1. We denote the persistence diagram corresponding to $\textrm{H}_k$ by ${{\,\textrm{dgm}}}_k$.

The original motivation for using persistent homology is to detect meaningful structures emerging in data. The simplest approach is by looking for points $p\in {{\textrm{dgm}}}_k$ far from the diagonal ($\textrm{d}=\textrm{b}$). These points represent cycles with a long lifetime ($\textrm{d}-\textrm{b}$), which are “significant”, whereas points near the diagonal are due to the noisy nature of the samples (see Fig. 1). While this approach is intuitive, justifying it theoretically and providing *quantitative statements*, are among the greatest challenges in the field. For geometric complexes, a strong case can be made^26^ that it is better to use the ratio $\pi = \textrm{d}/\textrm{b}$ to discriminate between signal and noise in ${\textrm{dgm}}_k$ ($k>0$), since the ratio $\pi$ is (a) scale invariant, and (b) more robust to outliers. For further discussion see “Methods”.

### Noise distribution in persistence diagrams

We assume that every persistence diagram can be decomposed into ${{\textrm{dgm}}}_k^{_{\textbf S}} \cup {{\textrm{dgm}}}_k^{_{\textbf N}}$, corresponding to the signal and noise parts. The signal corresponds to meaningful topological features which are latent in the data (e.g., points sampled near an annulus will always contain a hole in the middle). The noise part consists of features which are the result of randomness in the data. For formal definitions and a discussion, see Sect. 2 of the Supplementary Information. The fundamental challenge is to decide for each feature *p* in the diagram, whether $p\in {{\textrm{dgm}}}_k^{_{\textbf S}}$ or $p\in {{\textrm{dgm}}}_k^{_{\textbf N}}$, and provide quantitative guarantees on this decision. The distribution of ${{\textrm{dgm}}}_k^{_{\textbf N}}$ thus serves as a null-distribution, and revealing it would enable us to use powerful hypothesis testing methods.

The probabilistic analysis of ${{\textrm{dgm}}}_k^{_{\textbf{N}}}$ has been fruitful^22,23,25–27^, a more detailed discussion is provided in Sect. 1 of the Supplementary Information. However, while the mathematical theory is quite rich, translating it into statistical tools has lagged behind. The two main reasons are: (a) these results show that various limits exist, but in most cases without any explicit description, (b) these limits strongly depend on the underlying distribution. A more practical approach^13^models persistence diagrams using Gibbs measures, whose parameters can be estimated from the data. Due to the limitations imposed by the theoretical analysis, the statistical literature for *topological inference* is mostly based on the premise that the distribution of persistence diagrams is inaccessible. A prominent approach in this case is based on statistical bootstrap^18,19,28^. Other useful methods include distance to measures^29^, witness complexes^30^, and multi-cover bifiltrations^31,32^.

## Results

### The distribution of persistent cycles

Let $\mathcal {S}$ be a *d*-dimensional metric measure space, and let ${\textbf{X}}_n = (X_1,\ldots ,X_n)\in S^n$ be a sequence of random variables (points), whose joint probability law is denoted by $\mathbb {P}_n$. Let $\mathbb {P}= (\mathbb {P}_n)_{n=1}^\infty$, and denote $\mathbb {S}=(\mathcal {S},\mathbb {P})$, which we refer to as the *sampling model*. Fix a filtration type $\mathcal {T}$ (e.g., Čech or Rips), and a homological degree $k>0$, and consider the *k*-th *noise* persistence diagram ${{\textrm{dgm}}}_k^{_{\textbf{N}}}({\textbf{X}}_n;\mathcal {T})$, which in short we denote by ${{\textrm{dgm}}}_k$. We study the distribution of the random persistence values $\left\{ \pi (p)\right\} _{p\in {{\textrm{dgm}}}_k}$ (where $\pi (p) = {{\,\textrm{death}\,}}(p)/{{\,\textrm{birth}\,}}(p)$), and refer to them as the $\pi$-values of the diagram. Theoretical analysis shows that the largest $\pi$-value (${{\,\textrm{death}\,}}/{{\,\textrm{birth}\,}}$ ratio) of points in the noise (${{\textrm{dgm}}}_k^{_{\textbf{N}}}$) is $o((\log n)^{1/k})$^26^, while the $\pi$-values of the signal features (${{\textrm{dgm}}}_k^{_{\textbf{S}}}$) are $\Theta (n^{1/d})$^33^. Thus, the $\pi$-values provide a strong separation (asymptotically) between signal and noise in persistence diagrams. We stress that in this paper we study the **entire** ensemble of persistence values, not only the maximal ones.

#### Weak universality

**Figure 2:**
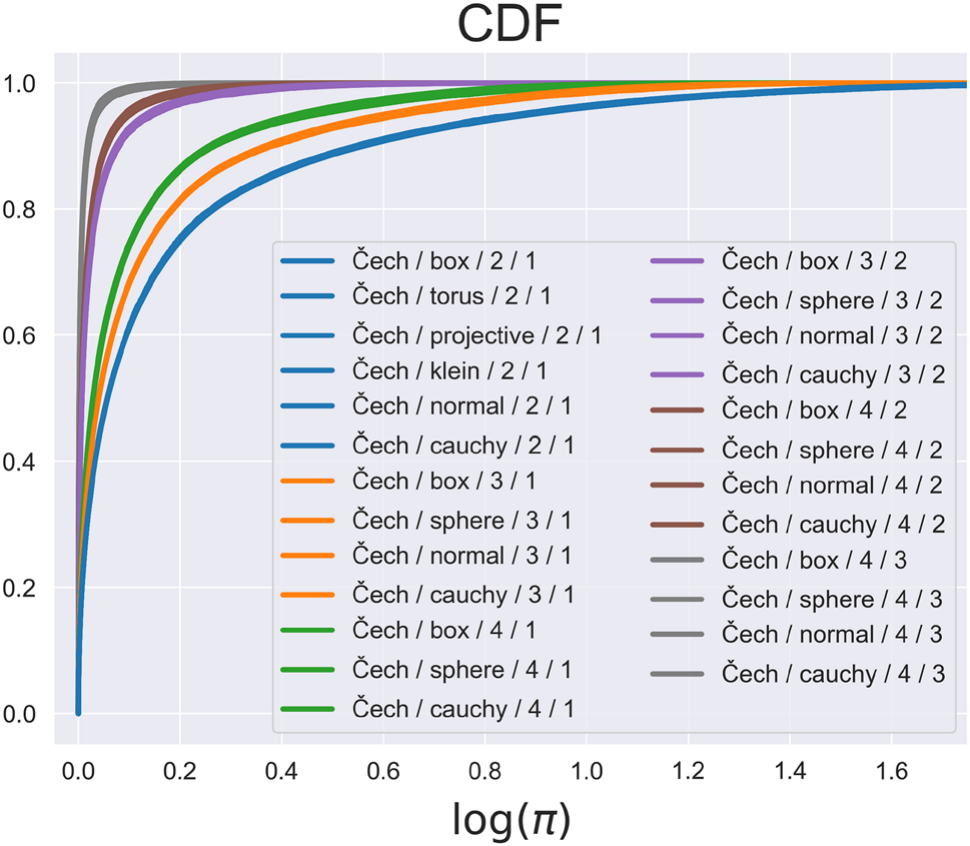
The distribution of $\pi$-values in the Čech complex. We take the empirical CDFs of the $\pi$-values (log-scale), computed from various iid samples. The legend format is $\mathcal {T}/ \mathbb {P}/ d / k$, where $\mathcal {T}$ is the complex type, $\mathbb {P}$ is the probability distribution, *d* is the dimension of the sampling space, and *k* is the degree of homology computed. By ‘box’, ‘torus’, ‘sphere’, ‘projective(-plane)’, and ‘Klein(-bottle)’ we refer to the uniform distribution on the respective space or its most natural parametrization, while ‘normal’ and ‘cauchy’ refer to the corresponding non-uniform distributions. See “Methods” and Sect. 3 in the Supplementary Information for further details.

We begin by considering the case where $\mathbb {P}_n$ is a product measure, and the points $X_1,\ldots ,X_n$ are iid (independent and identically distributed). Given ${{\textrm{dgm}}}_k$ as defined above, denote the empirical measure of *all* $\pi$-values

$$\begin{aligned} \Pi _{n} = \Pi _{n}(\mathbb {S},\mathcal {T},k):= \frac{1}{|{{\textrm{dgm}}}_k|}\sum _{p\in {{\textrm{dgm}}}_k}\delta _{\pi (p)}, \end{aligned}$$

where $\delta _x$ is the Dirac delta-measure at *x*. In Fig. 2 we present the CDF of $\Pi _{n}$ for the Čech complex with various choices of $\mathbb {S}$ and *k*. Similar plots are available for the Rips complex in Sect. 3 of the Supplementary Information. We observe that if we fix *d* (dimension of $\mathcal {S}$), $\mathcal {T}$, and *k*, then the resulting CDF depends on neither the space $\mathcal {S}$ nor the distribution $\mathbb {P}_n$. This leads to our first conjecture.

##### Conjecture 1

Fix $d,\mathcal {T},$ and $k>0$. For any $\mathbb {S}\in \mathcal {I}_d$,

$$\begin{aligned} \lim _{n\rightarrow \infty }\Pi _{n} = \Pi ^*_{d,\mathcal {T},k}, \end{aligned}$$

where $\Pi ^*_{d,\mathcal {T},k}$ is a probability distribution on $[1,\infty )$.

The precise notion of convergence and the extent of the class $\mathcal {I}_d$ are to be determined as future work. We conjecture that $\mathcal {I}_d$ is quite large. In our experiments, the space $\mathcal {S}$ varied across a wide range of manifolds and other spaces. The distribution $\mathbb {P}_n$ is continuous and iid, but otherwise fairly generic (possibly even without moments, see the Cauchy example in Fig. 2). We name this phenomenon “weak universality”, since on one hand the limit is independent of $\mathbb {S}$ (hence, universal), while on the other hand it does depend on $d,\mathcal {T},k$ and the iid assumption. This is in contrast to the results we discuss next.

#### Strong universality

**Figure 3:**
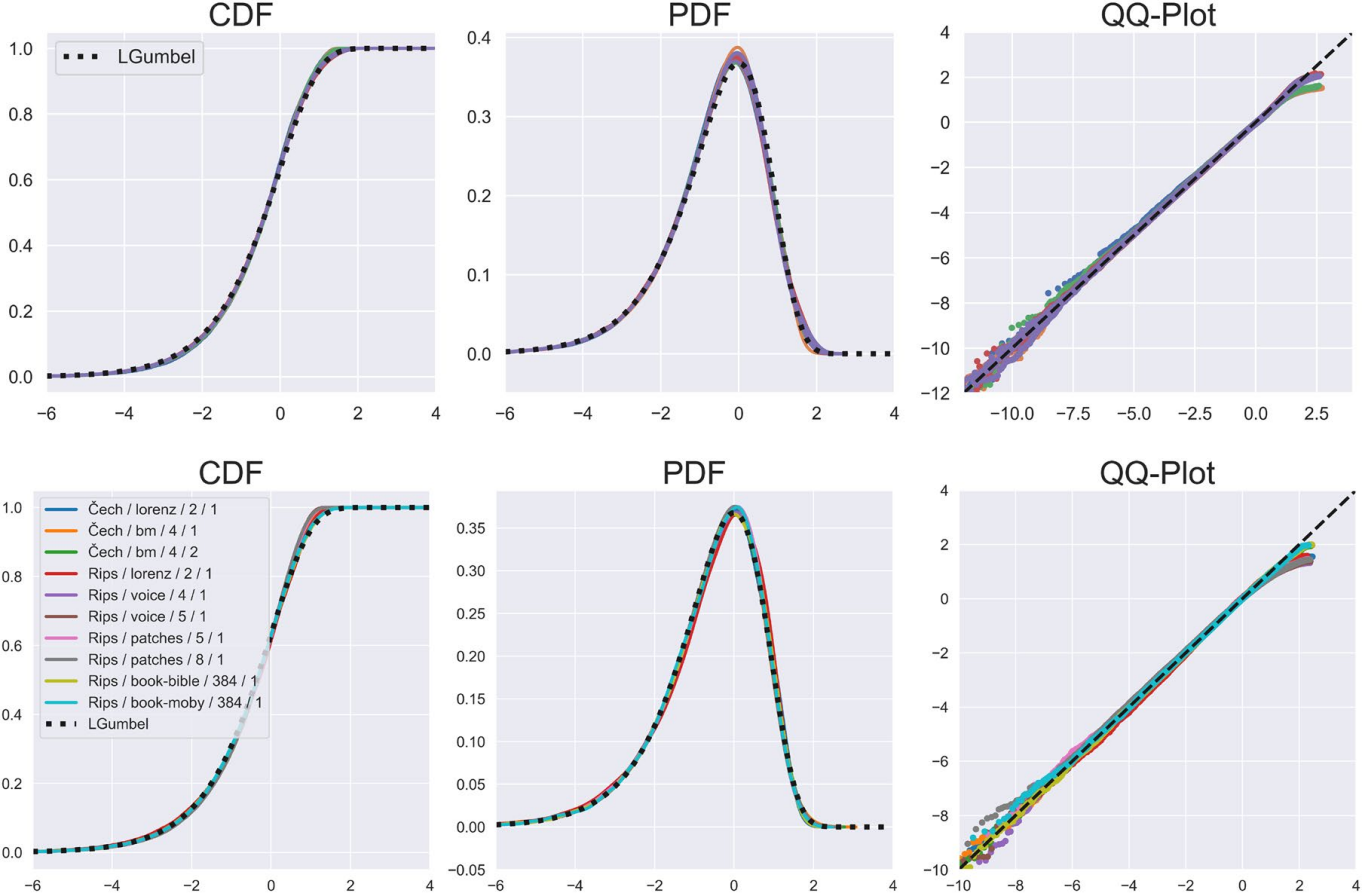
The distribution of $\ell$-values. (top) *All* the iid samples included in Fig. 2 (26 curves). (bottom) A selection non-iid and real-data point clouds. The left column shows the empirical CDF, and the middle column is the kernel density estimate for the PDF, with the LGumbel distribution shown as the dashed line. The right column shows the QQ plots compared this distribution.

The following procedure was discovered partly by chance. While non-intuitive, the results are striking. Given a random persistence diagram ${{\textrm{dgm}}}_k$, for each $p\in {{\textrm{dgm}}}_k$ apply the transformation

1 $$\begin{aligned} \ell (p) := A{{\,\mathrm{\log \log }\,}}(\pi (p)) + B, \end{aligned}$$

where

2 $$\begin{aligned} A = {\left\{ \begin{array}{ll}1 &{} \mathcal {T}=\text {Rips},\\ 1/2 &{} \mathcal {T}= \check{\textrm{C}}\text {ech},\end{array}\right. }\qquad B = -\lambda - A{\bar{L}}, \end{aligned}$$

and where ${\bar{L}} = \frac{1}{|{{\textrm{dgm}}}_k|} \sum _{p\in {{\textrm{dgm}}}_k}{{\,\mathrm{\log \log }\,}}(\pi (p))$ and $\lambda$ is the Euler-Mascheroni constant (= 0.5772156649$\ldots$). We refer to the set $\left\{ \ell (p)\right\} _{p\in {{\textrm{dgm}}}_k}$ as the $\ell$-values of the diagram. In Fig. 3 we present the empirical CDFs of the $\ell$-values, as well as the kernel density estimates for their PDFs, for all the iid samples that were included in Fig. 2. The plots for the Rips complex are similar, and can be found in Sect. 3 of the Supplementary Information. We observe that all the different settings ($\mathbb {S},\mathcal {T},k$) yield *exactly* the same distribution under the transformation given by (1). We refer to this phenomenon as “strong universality”.

While strong universality for iid point-clouds is by itself a very unexpected and useful behavior, a natural question is how generally it applies in other scenarios. In Fig. 3 we also include a selection of non-iid samples and real-data (see Experimental results and Methods for details). While the distribution of the $\pi$-values for these models is vastly different than the iid case, all of these examples exhibit the same strong universality behavior.

To summarize, our experiments highly indicate that persistent $\ell$-values have a universal limit for a wide class of sampling models $\mathbb {S}$, denoted by $\mathcal {U}$. For our main conjecture, we consider the empirical measure of *all* $\ell$-values,

$$\begin{aligned} \mathcal {L}_n = \mathcal {L}_n(\mathbb {S},\mathcal {T},k):= \frac{1}{|\textrm{dgm}_k|}\sum _{p\in {{\textrm{dgm}}}_k}\delta _{\ell (p)}. \end{aligned}$$

##### Conjecture 2

For any $\mathbb {S}\in \mathcal {U}$, $\mathcal {T}$, and $k\ge 1$,

$$\begin{aligned} \lim _{n\rightarrow \infty }\mathcal {L}_n = \mathcal {L}^*, \end{aligned}$$

where $\mathcal {L}^*$ is independent of $\mathbb {S}$, $\mathcal {T}$, and *k*.

Observe that in this Conjecture, the only dependence on the distribution generating the point-cloud is in the value of *B* (2) (similar to the role the mean and the variance play in the central limit theorem). In Sect. 5 of the Supplementary Information, we examine the value of *B* for different iid settings. As suggested by Conjecture 1, our experiments confirm that the value of *B* (for the iid case) depends on $d,\mathcal {T},k$, but is otherwise independent of $\mathbb {S}$. Revealing the exact relationship between all parameters remains future work.

##### Remark

We note that models with homogeneous spacing between the points, such as perturbed lattice models, or repulsive point processes, do not follow Conjecture 2. See Sect. 2.4 in the Supplementary Information.

#### A candidate distribution

A natural question is whether the observed limiting distribution $\mathcal {L}^*$ is a familiar one, and in particular, if it has a simple expression. Surprisingly, it seems that the answer might be yes. We denote the *left-skewed Gumbel distribution* by $\textrm{LGumbel}$, whose CDF and PDF are given by

3 $$\begin{aligned} F(x) = 1-e^{-e^x},\quad \text {and}\quad f(x) = e^{x-e^{x}}. \end{aligned}$$

The expected value of this distribution is the Euler-Mascheroni constant ($\lambda$) used in (2). In Fig. 3, the black dashed lines represent the CDF and PDF of the LGumbel distribution. In addition, the right column presents the QQ-plots of all the different models compared to the LGumbel distribution. These plots provide very strong evidence for the validity of our final conjecture.

##### Conjecture 3

$$\mathcal {L}^* = \textrm{LGumbel}$$.

##### Remark

The Gumbel distribution often emerges as the limit of extreme value distributions (i.e., minima or maxima). We wish to emphasize that the limiting LGumbel distribution in Conjecture 3 describes the **entire ensemble** of all $\ell$-values and so it **does not** describe any extreme values. Consequently, while there may exist an indirect connection to extreme value theory, it is by no means evident or straightforward. The appearance of the LGumbel distribution in this context, is therefore quite surprising.

##### Remark

The plots in Fig. 3 exhibit a remarkable fit with the LGumbel distribution, with one minor exception observed in the deviation of the tails of the QQ-plots. This slight deviation can be attributed to slow convergence rates. Existing theoretical results^26^ indicate that the largest $\pi$-value tends to infinity with *n*, but its growth rate is logarithmic. Consequently, the largest $\ell$-value is of order $\log \log \log (n)$, and so particularly the tails will exhibit a slow rate of convergence. Since the point-clouds we are able to process consist of at most millions of points, our ability to accurately capture the tail of the distribution is quite limited. It is important to note that this limitation holds regardless of the validity of Conjecture 3. While the limiting distribution has a non-compact support, our experiments cover a restricted range of death/birth values. Therefore, if we draw the QQ-plot against any other distribution with non-compact support, we will observe similar deviations.

### Experimental results

We present a large body of experimental evidence collected to support our conjectures. As the results do not seem to be significantly impacted by the choice of *n*, we leave this detail to the Supplementary Information (Section 3). Complementing the statistical plots presented here and in the Supplementary Information, we also performed a Kolmogorov-Smirnov goodness-of-fit test for our entire collection of point-clouds. The details are provided in Sect. 3.4 of the Supplementary Information. The test did not detect significant difference between the distribution of $\ell$-values and the LGumbel distribution in any of the point-clouds, providing further support for the validity of Conjectures 1, 2, 3.

#### Sampling from iid distributions

We began by considering samples from the uniform distribution on various compact manifolds, with diverse geometry and topology. Next, we tested non-uniform distributions in $\mathbb {R}^d$, by taking products of well-known distributions on $\mathbb {R}$. We attempted to test a wide range of settings. The beta distribution has a compact support ([0, 1]), while the normal and Cauchy distributions are supported on $\mathbb {R}$. The standard normal distribution is an obvious choice and Cauchy was chosen as a heavy-tailed distribution without moments. Finally, we considered more complex models—sampling from the configuration space of a closed five-linkage, and stratified spaces (intersecting manifolds of different dimensions). The results for many of the experiments are in Figs. 2 and 3 (see Sect. 3 of the Supplementary Information for the complete set of experiments). All of the iid sampling models we tested support Conjectures 1, 2, 3.

#### Non-iid distributions

To better understand the extent of universality, as well as to consider more realistic models, we tested more complex cases. We tested two vastly different models: sampling points from the path of a *d*-dimensional Brownian motion, and a discrete-time sample of the Lorenz dynamical system—a well-studied chaotic system. The results in Fig. 3 confirm that these non-iid models exhibit strong universality as well. Surprisingly, the results for the Brownian motion demonstrate the best fit with the LGumbel distribution, among all the settings we tested (see Figs. 10 and 11 in the Supplementary Information). This could be related to the fractal, or self-similarity, behavior of the Brownian motion, but remains a topic for future study.

#### Testing on real data

The most important test for Conjectures 2 and 3 is with real world data. We tested three different examples (see Methods for mode details). (1) Natural images: We sampled $3\times 3$ patches from natural gray-scale images taken from van Hateren and van der Schaaf dataset^34^. We applied the dimension reduction procedure proposed by Lee et al.^35^, which results in a point-cloud on a 7-dimensional sphere embedded in $\mathbb {R}^8$. We tested both the 7-dimensional point-cloud, as well as its lower-dimensional projections. (2) Audio recording: We applied the time-delay embedding transformation^36^ to an arbitrary speech recording to create a *d*-dimensional point-cloud. (3) Sentence embeddings: We used a pretrained sentence transformer^37^, to convert the entire text in a book into a 384-dimensional point-cloud. Our experiments using both the Čech and Rips complexes, show a remarkable matching to the universal distribution (see Fig. 3 for a subset).

### Application: hypothesis testing

Based on Conjectures 2–3, we present a hypothesis testing framework for individual cycles in persistence diagrams. We address finite and infinite cycles separately.

#### Finite cycles

Given a persistence diagram ${{\textrm{dgm}}}= \left\{ p_1,\ldots ,p_m\right\}$, our goal is to determine for each point $p_i$ whether it is signal or noise. This can be modelled as a multiple hypothesis testing problem with the *i*-th null-hypothesis, denoted $H_0^{(i)}$, is that $p_i$ is a noisy cycle. Assuming Conjectures 2 and 3, we can formalize the null hypothesis in terms of the $\ell$-values (1) as

$$\begin{aligned} H_0^{(i)}: \ell (p_i) \sim \textrm{LGumbel}. \end{aligned}$$

In other words, cycles that deviate significantly from the LGumbel distribution should be declared as *signal*. If the observed persistence $\ell$-value is *x*, then its corresponding *p*-value is computed via

4 $$\begin{aligned} p\text {-value}_i = \mathbb {P}\left( \ell (p_i) \ge x\;|\;H_0^{(i)}\right) = e^{-e^x}. \end{aligned}$$

Since we are testing multiple cycles simultaneously, we applied the Bonferroni correction to the *p*-values, which sufficed for our experiments. The signal part of a diagram (for significance level $\alpha$) can thus be recovered, via

$$\begin{aligned} {{\,{\textrm{dgm}}}}_k^{_{\textbf{S}}}(\alpha ) = \left\{ p\in {{{\textrm{dgm}}}}_k: e^{-e^{\ell (p)}} < \frac{\alpha }{|{{{\textrm{dgm}}}}_k|}\right\} . \end{aligned}$$

#### Infinite cycles

Computing persistent homology for an entire filtration is often intractable. The common practice is to fix a threshold $\tau$, and compute ${{\textrm{dgm}}}_k(\tau)$ for the partial filtration. This often introduces cycles that are “infinite”—i.e., born prior to $\tau$, but die after $\tau$. The question we address here is how to *efficiently* determine whether such cycles are statistically significant. Let $p=(\textrm{b},\textrm{d})\in {{\textrm{dgm}}}_k(\tau)$ be an infinite cycle, i.e., $\textrm{b}\le \tau$ is known and $\textrm{d}>\tau$ is *unknown*. While we do not know $\ell (p)$, we observe that $\ell (p)> \tau /\textrm{b}$, which gives an upper bound for the *p*-value,

$$\begin{aligned} p\text {-value}_i < p\text {-value}_i(\tau ):= e^{-e^{\tau /\textrm{b}}}. \end{aligned}$$

If $p\text {-value}_i(\tau )$ is below the required significance value (e.g. $\alpha /|{{\textrm{dgm}}}_k(\tau )|$), we can declare *p* as significant, despite not knowing the true death-time. Otherwise, we can determine the minimal value $\tau ^*$ required so that $p\text {-value}_i(\tau ^*)$ is below the significance value. We then compute ${{\textrm{dgm}}}_k(\tau ^*)$, and if the cycle represented by *p* remains infinite (i.e. $\textrm{d} > \tau ^*$), we declare it significant. We observe that for measuring significance, we do not need to know the exact value of $\textrm{d}$, only whether it is smaller or larger than $\tau ^*$, and we need only to compute the filtration up to $\tau ^*$, rather than the actual death time $\textrm{d}$. The key point is that the death time $\textrm{d}$ may be much larger than $\tau ^*$.

The procedure we just described works well for studying a single infinite cycle. However, it is likely that ${{\textrm{dgm}}}_k(\tau )$ contains multiple infinite cycles. Moreover, increasing the threshold may result in new infinite cycles emerging as well. We therefore propose the iterative procedure described in Algorithm 1. Briefly, at every step the algorithm picks one infinite cycle, and chooses the next threshold $\tau$ so that we can determine if it is significant or not. The value $\pi _{\min }(x)$ in the Algorithm 1, is the minimum $\pi$-value required so that the resulting *p*-value (4) is smaller than *x*. Formally,

$$\begin{aligned} \pi _{\min }(x) = \ell ^{-1}\left( F^{-1} \left( 1-x\right) \right) = \ell ^{-1}({{\,\mathrm{\log \log }\,}}(1/x)), \end{aligned}$$

where *F* is the CDF of the LGumbel distribution. In the algorithm, we choose the earliest-born infinite cycle ($\min (I)$), while we could have chosen the latest-born ($\max (I)$), or any intermediate value. This choice represents a trade-off between the number of iterations needed and the overestimation of $\tau$. Choosing the earliest born cycle results in the smallest threshold, but with potentially more iterations, while choosing the last cycle will have fewer iterations with a possible overestimation of $\tau$.

**Table:** 

**Algorithm 1** Finding the threshold for infinite cycles	
$$\begin{aligned} & \tau \leftarrow \tau_{0} \\ & {\mathbf{do}} \\ & \quad D \leftarrow {\text{dgm}}_{k} (\tau ) \\ & \quad I \leftarrow \left\{ {{\text{b}}:({\text{b}},{\text{d}}) \in D,\,{\text{d}} = \infty ,{\text{and}}\,\tau /{\text{b}} < \pi_{{{\text{min}}}} \left( {\alpha /\left| D \right|} \right)} \right\} \\ & \quad \tau \leftarrow \left\{ {\begin{array}{*{20}l} {\min \,(I) \cdot \pi_{\min } \left( {\alpha /\left| D \right|} \right)} \hfill & {I \ne \emptyset } \hfill \\ \tau \hfill & {I = \emptyset } \hfill \\ \end{array} } \right. \\ & {\mathbf{while}}\,\left| I \right| > 0 \\ & {\mathbf{return}}\,\tau \\ \end{aligned}$$	

#### Examples

We present two examples for our hypothesis testing framework. In all our experiments, we set the desired significance level to be $\alpha =0.05$.

**Figure 4:**
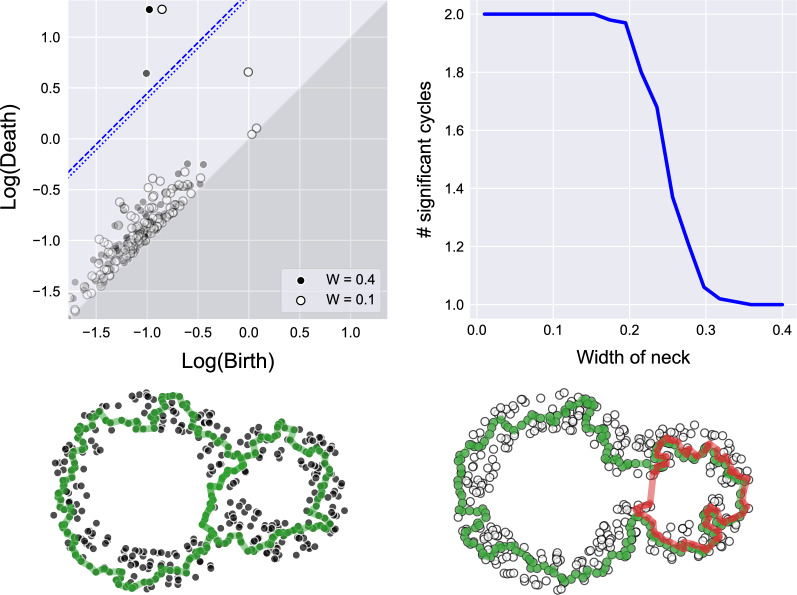
Computing *p*-values for the 8-shape (a wedge of circles). (top-left) Persistence diagrams for two instances of the 8-shape with different neck gaps, with the significance lines shown for $\alpha =0.05$ (the dotted and dashed lines correspond to $W=0.1$ and $W=0.4$, respectively). (top-right) The average number of signal cycles detected in 100 repetitions, as a function of the neck gap. (bottom) In green we show significant cycles. On the left ($W=0.1$) we see two significant cycles (*p*-values = 0.0005, 0.012), and on the right ($W=0.4$) only the outer cycle is significant (*p*-value=0.0015).

- **Computing p-values:** We begin with a toy example, sampling 1000 points on an 8-shape (a wedge of circles) in $\mathbb {R}^2$ (see Fig. 4), where we vary the width of the neck. We expect one cycle to always be significant (the outer one), but the significance of the second cycle depends on the width of the neck. For each width value we computed the persistence diagram, and checked how many cycles were significant (i.e., $p\text {-value}<\frac{\alpha }{|{{\textrm{dgm}_k}}|}$). The results are presented in Fig. 4. For very small neck widths, our sample is indistinguishable from the 8-shape where the neck is fully connected, and hence both cycles are detected.

**Figure 5:**
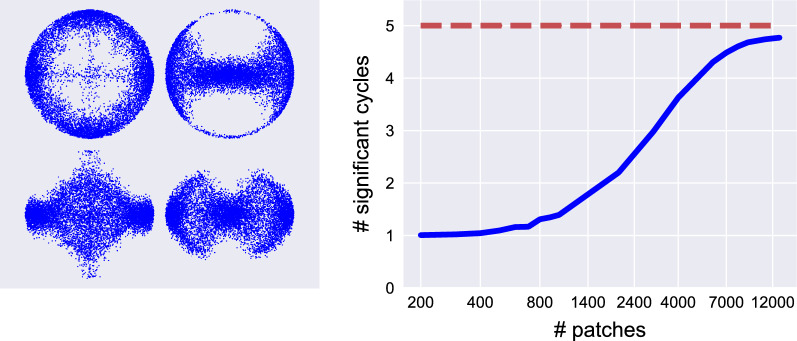
Testing the 3-circle model in the natural image patches. (left) Four different 2-dimensional projections of the 8-dimensional patches point-cloud. The projection on the top-right shows a 1-cycle that is quite “thin” and contains large gaps. (right) The *p*-value curve for the patches dataset as a function of the number of samples. We observe that we are not detecting 5-cycles in 100% of the cases. This is most likely due to the “thin” cycle.

Next, we apply this method to a real-world dataset, specifically the van-Hateren natural image database mentioned earlier. The main claim by de-Silva & Carlsson^30^ is that the space of $3\times 3$ patches has a 3-circle structure in $\mathbb {R}^8$, leading to the conclusion that the patches are concentrated around a Klein-bottle^38^. This was supported by five relatively long 1-cycles in the persistence diagram computed over the patches. To provide quantitative statistical support for this claim, we randomly selected a subset of the patches, processed them^30^, and computed *p*-value for all cycles (using the Rips complex). We repeated this experiment for varying numbers of patches, and computed the average number of detected signal cycles over 250 trials. The results are presented in Fig. 5. Firstly, we observe that there exists a single 1-cycle that is nearly always detected (the primary circle), while other cycles appear as we increase the sample size. Secondly, we observe that the fifth cycle is intermittently detected. Plotting a 2-dimensional projection of the points, we see that this cycle contains very sparse areas, increasing its birth time and consequently the *p*-value.

To conclude, using this approach, we are able to correctly detect the signal cycles discovered by de-Silva & Carlsson^30^, as well as *quantitatively declare the significance level* for each cycle.

- **Infinite cycles:** To test Algorithm 1, we used a point-cloud on a 2-dimensional torus in $\mathbb {R}^3$. Computing the *p*-values of the two signal 1-cycles requires a massive complex and becomes computationally intractable for even a few thousand points. Using Algorithm 1, the filtration size is incrementally increased until the signal was detected. At 50k points, this saves approximately 80% of the edges that would otherwise be needed. This ratio would significantly increase for higher dimensional simplexes. See Sect. 7 in the Supplementary Information for the complete details.

## Discussion

In this work, we revealed a highly surprising phenomena: viewed through the lens of the $\ell$-values, almost all persistence diagrams follow the same universal distribution. This claim is supported by an exhaustive set of experimental results, ranging from iid samples to real data. We expect two major outcomes of this paper: (1) revealing the full extent of Conjectures 1, 2, 3 will provide fertile grounds for a whole new line of theoretical research in stochastic topology; (2) the universality properties enable the development of powerful statistical tools in TDA with hypothesis testing being only the tip of the iceberg. For example, the conjectures may lead to stronger bounds on distances between persistence diagrams coming from data, or further algorithmic advances based on the universal distribution.

## Methods

### Persistent homology

In order to turn point-clouds into shapes that can be studied topologically, the common practice in TDA is to use their geometry to generate simplicial complexes. A simplicial complex can be thought of as a “high-dimensional graph” where in addition to vertices and edges, we include triangles, tetrahedra, and higher dimensional simplexes. Formally, we say that *X* is an *abstract simplicial complex* over a set *S*, if *X* consists of finite nonempty subsets of *S*, and is closed under inclusion (i.e., $A\in X$ and $B\subset A$ implies $B\in X$). We refer to elements in *X* of size $k+1$ as either *k*-simplexes or *k*-faces.

For every simplicial complex *X* we can compute its *homology groups*, denoted $\textrm{H}_k(X)$. Homology is a classical construction in algebraic topology^39^, describing the structure of simplicial complexes in terms of *chains*. Chains are formal linear combinations of simplexes of the same dimension – $\sum _i \lambda _i \sigma _i$, where $\sigma _i$ are *k*-simplexes, and $\lambda _i$ are some coefficients. Commonly, the coefficients are taken in a finite field (e.g., ${\mathbb {Z}}_2$), in which case, the space of *k*-chains forms a vector space, denoted $C_k$. The key ingredient in defining homology is the boundary operator $\partial _k: C_k\rightarrow C_{k-1}$. This is a linear map describing how $(k-1)$-simplexes are attached to *k*-simplexes. Elements in the kernel of $\partial _k$ are called *k*-cycles (i.e., chains with zero boundary), while elements in the image of $\partial _{k+1}$ are called *k*-boundaries. The *k*-th homology is then defined as the space of all *k*-cycles which are not *k*-boundaries, formally given as the quotient $\textrm{H}_k = \textrm{ker}\;\partial _k / \textrm{im}\; \partial _{k+1}$.

Loosely speaking, the homology groups $\textrm{H}_k(X)$ ($k\ge 0$) represent structural information about the complex *X*. For example, the basis of $\textrm{H}_0(X)$ corresponds to the connected components, the basis of $\textrm{H}_1(X)$—to closed loops surrounding holes, and the basis of $\textrm{H}_2(X)$—to closed surfaces surrounding cavities (can be thought of as “bubbles” or “air-pockets”). For a more formal definitions and background, we refer the reader to the literature on algebraic topology^39,40^.

In this paper we are interested in *geometric complexes*, i.e., abstract simplicial complexes whose vertex set is a point-cloud, and simplexes are determined by the geometric configuration of the points. The two most common constructions in TDA are both parameterized by a scale parameter (radius) $r>0$, and defined as follows:

- **Vietoris-Rips complex:** We include a subset of $(k+1)$ points as a *k*-simplex, if all points are within distance *r* from each other.
- **Čech complex:** We place a ball of radius *r* around each point, and then include a subset of $(k+1)$ points as a *k*-simplex, if the intersection of the corresponding balls is non-empty.

See Fig. 2 in the Supplementary Information for examples. Denoting by $X_r$ either of the above complexes, an important observation is that this construction is “increasing”, in the sense that for $r<s$, we have $X_r\subseteq X_{s}$. Such sequences are also known as *filtrations*. While we can study the homology of each individual complex $X_r$ separately, a more powerful approach is to consider the entire range of parameters (in this case $r\in [0,\infty )$) and track the *evolution* of $\textrm{H}_k(X_r)$ along this range. A key property of homology is that it is *functorial*, i.e., every map between simplicial complexes induces a linear map between their respective homology groups. Using the inclusion maps $i:X_r\hookrightarrow X_s$, we have the corresponding linear maps in homology $i_*:\textrm{H}_k(X_r)\rightarrow \textrm{H}_k(X_s)$ for all $r<s$. *Persistent homology﻿*^41,42^ uses this collection of linear maps to describe how homology groups change over the filtration. This is done by assembling all the homology groups and the maps between them into an algebraic structure called a *persistence module*. A key result in topological data analysis^43^ is that persistence modules can be *uniquely* decomposed into a direct-sum of “basis-like” elements. These elements correspond to *k*-cycles that appear (born) at some parameter value, and disappear (die) at a later value in the filtration. Given this decomposition, we can summarize the structure of the persistence module, using a collection of points $(\textrm{b},\textrm{d})\in \mathbb {R}^2$, corresponding to the birth and death times of these basis elements. This representation is known as a *persistence diagram* (see Fig. 1). Persistent homology has been extensively studied, and employed in numerous applications. 

### Multiplicative persistence

**Figure 6:**
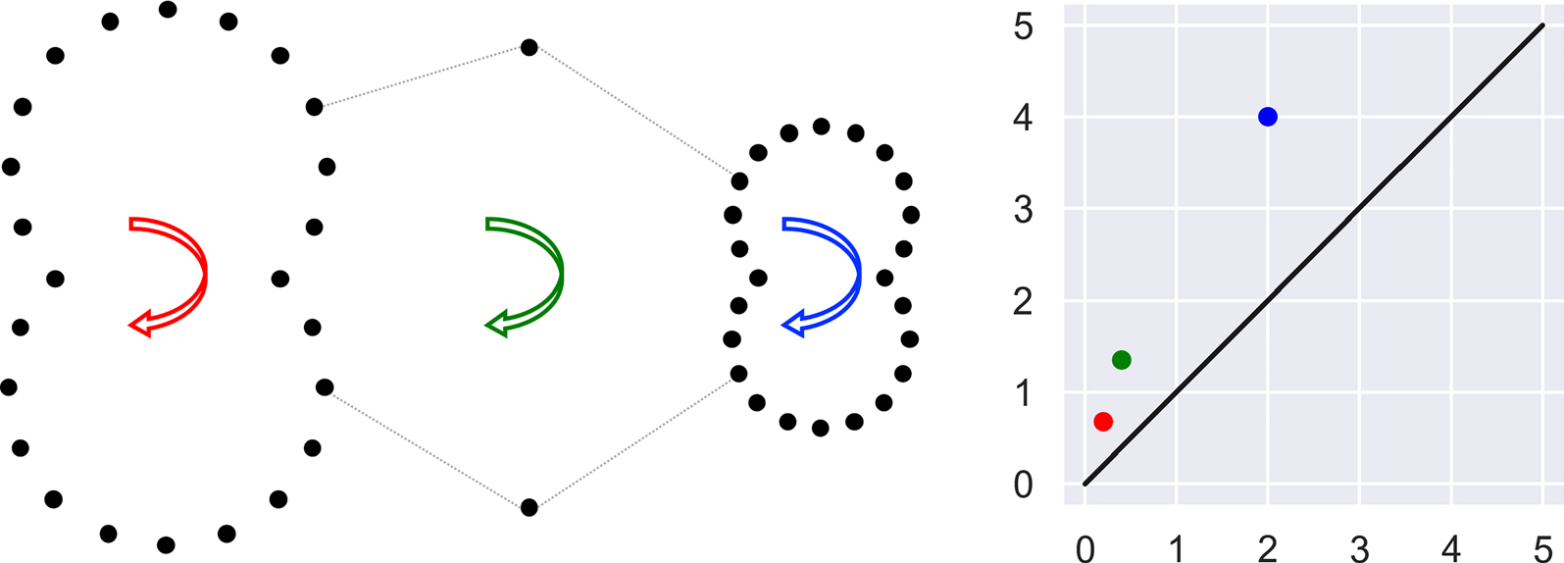
Motivation for multiplicative persistence. The point-cloud on the left generates three 1-dimensional cycles (marked by the arrows). On the right we see the corresponding persistence diagram for the Čech filtration with matching cycle colors. The coordinates of the points are $p_1 = (0.2, 0.675)$ (red), $p_2 = (0.4, 1.35)$ (green), and $p_3 = (2,4)$ (blue). Their corresponding lifetimes are $\Delta _1 = 0.475$, $\Delta _2 = 0.95$, and $\Delta _3 = 2$. This might lead us to declare the blue cycle as the most significant, while it seems to be generated by outliers. In addition, while the structure of the other two cycles (red and green) is identical, their lifetime is different. Both issues are resolved if we take the death/birth ratio instead. In this case, we have $\pi _1 = \pi _2 = 3.375$, while $\pi _3 = 2$.

The significance of topological features is often measured by the lifetimes of persistent cycles, i.e., $\Delta = ({{\,\textrm{death}\,}}-{{\,\textrm{birth}\,}})$. This method is intuitive in toy examples (see Fig. 1), as it captures the geometric “size” of topological features. However, a strong case can be made^26^ that for geometric complexes the ratio

$$\begin{aligned} \pi = ({{\,\textrm{death}\,}}/{{\,\textrm{birth}\,}}) \end{aligned}$$

is in fact a more robust statistic to discriminate between signal and noise in persistence diagrams (for $k>0$). There are two main justifications for this statement. Firstly, the ratio $\pi$ is scale invariant, so that cycles that have exactly the same structure but exist at different scales are weighed the same. Secondly, datasets often contain outliers that may generate cycles with a large diameter, and consequently their lifetime $\Delta$ will also be large. However, the value of $\pi$ for such outliers should remain low, compared to features that occur in dense regions (see Fig. 6).

In previous work^26^, the asymptotic scaling for the largest $\pi$-value was studied. Denoting $\pi _{k}^{\max } := \max _{p\in {{\textrm{dgm}}}_k} \pi (p)$, the main result (Theorem 3.1) shows that with high probability

$$\begin{aligned} A\left( \frac{\log n}{{{\,\mathrm{\log \log }\,}}n}\right) ^{1/k} \le \pi _{k}^{\max } \le B \left( \frac{\log n}{{{\,\mathrm{\log \log }\,}}n}\right) ^{1/k}, \end{aligned}$$

for some constants $A,B>0$. This is in contrast to *signal* cycles, who we know^33^ are of the order of $n^{1/d}$. Thus, the $\pi$-values provide a strong separation (asymptotically) between signal and noise in persistence diagrams.

#### Remark

These previous results are phrased for special cases (i.e., the uniform distribution on a *d*-dimensional box^26^ and a *d*-dimensional flat torus^33^). Nevertheless, the proofs can be adapted to a wide class of compact spaces and distributions, with the same asymptotic rates.

### Experimented point-clouds

In this section we provide the details on the models generating the various point-clouds tested. Tables summarizing all the tested settings, as well as the full results, are available in Sect. 3 of the Supplementary Information. We first discuss a few methodological points with respect to the experiments.

1. Most experiments on the Čech complex (particularly in 2 or 3-dimensions), were done in practice using the much more computationally efficient Delaunay-Alpha complex (especially for lower dimensions), which has an identical persistence diagram.
2. The experiments were run primarily on the Apocrita HPC cluster, using several different software packages including Gudhi^44^, Ripser^45^, Eirene^46^, Dionysus^47^, and Diode^48^.
3. The persistence diagrams for these experiments and the associated code can be found at can be found at https://doi.org/10.7910/DVN/CNIHYV and https://github.com/primozskraba/PersistenceUniversality respectively.

#### Remark

In all the considered settings, it is necessary to assume that the diagrams we analyze consist only of noisy cycles, without any signal. This assumption is essential, particularly when estimating the CDF or the value of the normalization parameter *B*. In cases where the signal is known (e.g., an annulus), we can manually exclude the signal features. However, in cases where the signal is unknown (e.g., audio sampling, sentence embeddings), we cannot do so. Nevertheless this issue is not significant in practice. The diagrams we generate typically contain thousands to millions of points. Thus, unless we anticipate a massive amount of signal features, the inclusion of signal cycles in our estimates will have a negligible effect, especially in the ${{\,\mathrm{\log \log }\,}}$ scale. This observation is particularly important for the applicability of our hypothesis testing framework.

#### Sampling from iid distributions

The first setting tested is where points are sampled from a random model in an iid fashion. Let $X = (X^{(1)},\ldots , X^{(d)})$ denote a single point in one of the iid samples. We describe briefly how *X* is generated in each of the models.

- **Box:** *X* is uniformly distributed in $[0,1]^d$.
- **Ball:** *X* is uniformly distributed in a unit *d*-dimensional ball. Sampling was done using the rejection-sampling method.
- **Annulus:** *X* is uniformly distributed in a *d*-dimensional annulus with radii in the range [1/2, 1]. Sampling was done using the rejection-sampling method.
- **Sphere:** *X* is uniformly distributed on a *d*-dimensional unit sphere, embedded in $\mathbb {R}^{d+1}$. Sampling was done by generating a standard ($d+1$)-dimensional normal variable, and projecting it on the unit sphere.
- **Beta(a, b):** The coordinates $X^{(1)},\ldots , X^{(d)}$ are sampled independently from the Beta(a,b) distribution.
- **Cauchy:** The coordinates $X^{(1)},\ldots , X^{(d)}$ are sampled independently from the Cauchy distribution.
- **Normal:** The coordinates $X^{(1)},\ldots , X^{(d)}$ are sampled independently from the standard normal distribution.
- **Torus:** We generate points on the 2-dimensional torus embedded in $\mathbb {R}^3$ as follows. We generate two independent variables $\phi$ and $\theta$ uniformly in $[0,2\pi ]$. Then we take the coordinates to be:
  $$\begin{aligned} X^{(1)} = (R_1+R_2\cos (\phi ))\cos (\theta ), \quad X^{(2)} = (R_1+R_2\cos (\phi ))\sin (\theta ),\quad X^{(3)} = R_2\sin (\phi ). \end{aligned}$$
  We used $R_1=2$ and $R_1=1$.
- **Klein:** We sample an embedding of the Klein bottle into ${\mathbb {R}}^4$ as follows. We generate two independent variables $\phi$ and $\theta$ uniformly in $[0,2\pi ]$. The value of *X* is then computed as
  $$\begin{aligned} \begin{array}{ll} X^{(1)} = (1+\cos (\theta ))\cos (\phi ), &{}\quad X^{(2)} = (1+\cos (\theta ))\sin (\phi ), \\ X^{(3)} = \sin (\theta )\cos \left( \frac{\phi }{2}\right), &{} \quad X^{(4)} = \sin (\theta )\sin \left( \frac{\phi }{2}\right). \end{array} \end{aligned}$$
- **Projective:** We sample an embedding of the real projective plane into ${\mathbb {R}}^4$. We generate independent variables $U_0$, $V_0$ and $W_0$ from the standard normal distribution. Next we take $(U, V, W) = \frac{(U_0,V_0,W_0)}{\sqrt{U_0^2+V_0^2+W_0^2}}\in \mathbb {S}^2$, and define
  $$\begin{aligned} X^{(1)} = UV, \quad X^{(2)} = UW, \quad X^{(3)} = V^2 - W^2, \quad X^{(4)} = 2VW. \end{aligned}$$
- **Linkage:** This model samples the configuration space of unit pentagonal linkages, i.e. a pentagon where adjacent edges are of unit length. To sample this space, we first fix two vertices at $p_1=(0,0)$ and $p_2=(1,0)$ respectively. Next, we generate two independent variables $\phi$ and $\theta$ uniformly in $[0,2\pi ]$, and set
  $$\begin{aligned} p_5=(\cos (\phi ),\sin (\phi )), \quad p_3=(1+\cos (\theta ),\sin (\theta )). \end{aligned}$$
  If $\Vert p_3-p_5\Vert >2$, the sample is rejected as there is no linkage with the chosen angles. Otherwise, there are two possible choices of the last point $p_4$. Let $(q^{(1)},q^{(2)})$ denote the midpoint of $\overline{p_3 p_5}$. Then
  $$\begin{aligned} p_4 = q + S \frac{\sqrt{1-\Vert q-p_5\Vert ^2}}{\Vert q-p_5\Vert } \left( p_5^{(2)} -q^{(2)},q^{(1)}-p_5^{(2)} \right) \end{aligned}$$
  where *S* is independent of $\phi ,\theta$, and ${\mathbb {P}}(S=1) = \frac{1}{2}$ and ${\mathbb {P}}(S=-1) = \frac{1}{2}$. See Fig. 1 in the Supplementary Information for an example.
- **Neptune:** We construct a sample from the surface of the statue of Neptune. We used a triangulation of the surface^49^, consisting of 4,007,872 triangles. To generate a sample, we first compute the area of each triangle and then choose a triangle at random with probability inversely proportional to the area of the triangle. We then pick a point uniformly in the chosen triangle. See Fig. 1 in the Supplementary Information.
- **Hennenberg:** We construct a sample of the Henneberg surface in ${\mathbb {R}}^3$ (see Fig. 1 in the Supplementary Information). We start by generating two independent variables $\phi$ and $\theta$ uniformly in $[0,2\pi ]$. We then construct the sample by
  $$\begin{aligned} X^{(1)}&= 2\cos (\theta )\sinh (\phi ) - \frac{2}{3}\cos (3\theta )\sinh (3\phi ),\\ X^{(2)}&= 2\sin (\theta )\sinh (\phi ) + \frac{2}{3}\sin (3\theta )\sinh (3\phi ),\\ X^{(3)}&= 2\cos (2\theta )\cosh (2\phi ). \end{aligned}$$
- **Stratified spaces:** To construct *X*, we consider two spaces $M_1 \subset M_2$ such that the dimension of $M_1$ is less than $M_2$. Then one of the two spaces is chosen with some probability *p* (which is a parameter of the model), and the chosen space is sampled uniformly. Although several models were tried, in the examples we show a plane $[-1,1]^2$ embedded in the middle of a cube $[-1,1]^3$. In other words, if the point (*x*, *y*) is chosen from the plane, the coordinates would be (*x*, *y*, 0).

#### Sampling from non-iid distributions

In addition to the iid setting, we also tested two examples of non-iid point-clouds. The first example is sampling the path of a *d*-dimensional Brownian motion $W_t$. To sample $W_t$ at times $t=1,\ldots ,n$ we use the fact that $W_t$ has stationary independent increments. We start by taking $Z_1,\ldots ,Z_n$ to be iid *d*-dimensional standard normal variables, and then we define $X_i = W_{t=i}= \sum _{i=1}^n Z_i$.

The second example we examined is a discrete-time sample of the Lorenz dynamical system, which is generated as follows. We start by picking a random initial point, uniformly in $[0,1]^3$. We then generate the sample using the differential equations,

$$\begin{aligned} \frac{dX^{(1)}}{dt}&= \sigma \left( X^{(2)} - X^{(1)}\right) ,\\ \frac{dX^{(2)}}{dt}&= X^{(1)} \left( \rho - X^{(3)}\right) - X^{(2)},\\ \frac{dX^{(3)}}{dt}&= X^{(1)}X^{(2)} - \beta X^{(3)}. \end{aligned}$$

We use $\sigma =45$, $\rho = 54$, and $\beta =10$, and a numerical approximation with $dt=0.1$ to generate a trajectory for the number of samples required. Note that each instance is a single trajectory. See Fig. 9 in the Supplementary Information for examples.

#### Testing on real data

Finally, we tested the strong-universality conjectures against two examples of real data—patches of natural images, and sliding windows of voice recordings. In this section we provide more details about these examples.

$\bullet$ **Image patches: **The images were taken from van Hateren and van der Schaaf^34^ database. This database contains a collection of about 4,000 gray-scale images. We follow the procedure described in Lee et al.^35^. We randomly select patches of size $3\times 3$ from the entire dataset. This gives us a point-cloud in $\mathbb {R}^9$. Let $\textbf{x}= (x_1,\ldots ,x_9)\in \mathbb {R}^9$ represent the log-values of a single patch, and follow the following steps:

1. Compute the average pixel value ${\bar{\textbf{x}}}$, and subtract it from all 9 pixels in the patch, i.e. $\textbf{y}= \textbf{x}-{\bar{\textbf{x}}}$
2. Compute the “D-norm” (a measure for contrast), $\Vert \textbf{y}\Vert _D = \sqrt{\textbf{y}^TD\textbf{y}}$.
3. Use the D-norm to normalize the pixel values, $\textbf{z}= \textbf{y}/ \left\| \textbf{y}\right\| _D$.
4. Use the Discrete Cosine Transform (DCT) basis to change the coordinate system, $\textbf{v}= \Lambda A^T \textbf{z}$.

The values of $D,A,\Lambda$, as well as more details about this proecedure can be found in the literature^35,38^. The process above results in a point-cloud lying on the unit 7-dimensional sphere in $\mathbb {R}^8$.

For the topological analysis^38^, the point-cloud is further filtered, in order to focus on the “essential” information captured by the patches. This is done in two steps:

1. Keep only “high-contrast” patches—whose D-norm is in the top 20%.
2. Compute the distance of each of the remaining patches to their k-nearest neighbor (with $k=15$), and keep only the patches in the bottom 15%.

We repeat the exact procedure performed Carlsson et al.^38^ for two main reasons. Firstly, we wanted to test our hypothesis testing framework to assign *p*-values to the cycles found using this procedure. Secondly, this procedure makes the sample distribution more intricate, and adds dependency between the sample points. We wanted to challenge our conjectures with data as complex as possible. In addition to taking the original 8-dimensional point-cloud, we also examined its lower dimensional projections for dimensions $d=3,\ldots ,7$. The sample size used was $n=50,000$ for all dimensions.

- **Sound recordings:** We took an arbitrary audio recording (the voice of one of the authors), and applied the time-delay embedding transformation^36^ to convert the temporal signal into a *d*-dimensional point-cloud. The voice recording is a 20 seconds excerpt, sampled at 16KHz, 48Kbps. We denote the corresponding discrete time signal as $V_t$. To convert the signal into a point-cloud we used the following:
  $$\begin{aligned} X_i = (V_{i\Delta }, V_{i\Delta +\tau },\ldots , V_{i\Delta +(d-1)\tau }) \in \mathbb {R}^d,\quad i=1,\ldots ,n. \end{aligned}$$
  The values we took for the example here were $\Delta = 3$, and $\tau = 7$. This, in particular, generates overlap between the windows, which guarantees strong dependency both between the points, and between the coordinates of each point. The sample size taken was $n=50,000$.
- **Sentence embeddings: **Choosing several freely available texts of sufficient length—we chose the Bible, Ulysses by James Joyce, and Moby Dick by Herman Melville, obtained from Project Gutenberg. Each book was first tokenized into sentences using the Natural Language Toolkit (NLTK)^50^. Each sentence was then embedded into a 384 dimensional space using a sentence transformer. These are pretrained models which can be used to compute the embeddings. We used the **all-MiniLM-L6-v2** model^37^. This model has been trained on over a billion sentences. The persistence diagram was then computed for resulting point cloud using Euclidean distance. The texts were chosen to so that they generated between 9000-30,000 sentences.

## Supplementary Information

Supplementary Information.

## Data Availability

The experimental data and code for all the results shown can be found at https://github.com/primozskraba/PersistenceUniversality and https://doi.org/10.7910/DVN/CNIHYV, respectively.
