# Supplementary material for: A universal null-distribution for topological data analysis
Source: Sci Rep. 2023 Jul 28;13:12274. doi: 10.1038/s41598-023-37842-2 (PMC10382541; doi:10.1038/s41598-023-37842-2)
Supplement: Supplementary file 1 — Supplementary Information. [file 41598_2023_37842_MOESM1_ESM.pdf]

A UNIVERSAL NULL-DISTRIBUTION FOR TOPOLOGICAL DATA ANALYSIS – SUPPLEMENTARY MATERIAL

OMER BOBROWSKI[1,2] AND PRIMOZ SKRABA[2]

1. Detailed Previous Work

We first begin with a more complete review of relevant applications and related work. Persistent homology has been applied successfully in numerous applications developed over the last decade, in areas such as neuroscience [5, 27, 29, 44], medicine and biology [15, 21, 37, 38], material sciences [31, 34, 36, 40, 43], dynamical systems [35, 52, 55], and cosmology [3, 45, 54]. A fundamental challenge in these applications is to determine quantitatively how significant topological features are. In the above work, this is often done qualitatively or with respect to an application-specific hypothesis/model rather than a general methodology.

From a theoretical point of view, the study closest in spirit to our current work is [32], where persistence diagrams were considered as random measures. The main result shows that for a wide class of stationary processes there exists a non-random limiting measure. Later [25] proved that these measures have density, and [48] extended the limit to marked point processes. In [42] the limit of persistence diagrams as compact sets was studied, in an extreme value setting. Other related works consider various marginals of persistence diagrams (e.g., Betti numbers, Euler characteristic) and study their limiting behavior in various settings [7, 8, 10, 11, 33, 41, 56, 57]. While these results have established a rich mathematical theory for persistence diagrams, translating them into statistical tools has lagged behind. The two main reasons are: (a) these results show that various limits exist, but in most cases without any explicit description, (b) these limits are highly dependent on the distribution generating the point-cloud.

Quite a different approach was taken in [3], where persistence diagrams were modelled as Gibbs measures, whose parameters can be estimated from the data. Once the model parameters are estimated, one can use MCMC algorithms to generate synthetic persistence diagrams. Generating random diagrams allows, for example, to compare different diagrams via hypothesis testing. This framework is quite generic and powerful and was further developed in [43]. However in this type of approach, the resulting distribution varies between different point-clouds, and the parameters need to be estimated for every model separately.

With respect to topological inference, the TDA literature provides various powerful methods, all based on the premise that the distribution of persistence diagram is inaccessible. In [26] the authors introduced confidence intervals for persistence diagrams, overcoming this issue by using sub-sampling methods and the stability of the persistence diagrams [22]. The confidence intervals are computed for an entire diagram, rather than for individual features, and thus are quite coarse. In addition, this approach requires restrictive assumptions on the underlying space (closed manifold), as well as the probability distributions and its derivatives. Other useful methods include distance to measures [17, 18], witness complexes [24], persistence landscapes [13] and other vectorization techniques [2, 14, 38, 46] , estimated null distributions [21], multi-cover bifiltrations [23, 47], and other sub-sampling based methods [6, 4, 19, 18, 50, 1].

2. Signal vs. Noise

In this section, we provide more details on the assumed decomposition of persistence diagrams into the signal and noise parts. Figure 1 presents a random sample from a figure eight or a wedge of two annuli in $\mathbb{R}^2$ (left), with the corresponding H_1 persistence diagram (right). The signal part of this diagram consists of the two points far above the diagonal, corresponding to the two holes in the figure eight. All other points are much closer to the diagonal, and arise due to the randomness of the sampling, rather than the underlying structure of the space. We therefore consider them as the noise. Note that while the signal points will appear in a different locations if we re-sample the shape, the *stability theorem* for persistence diagrams [22] ensures that the signal points across different samples will be close to each other.

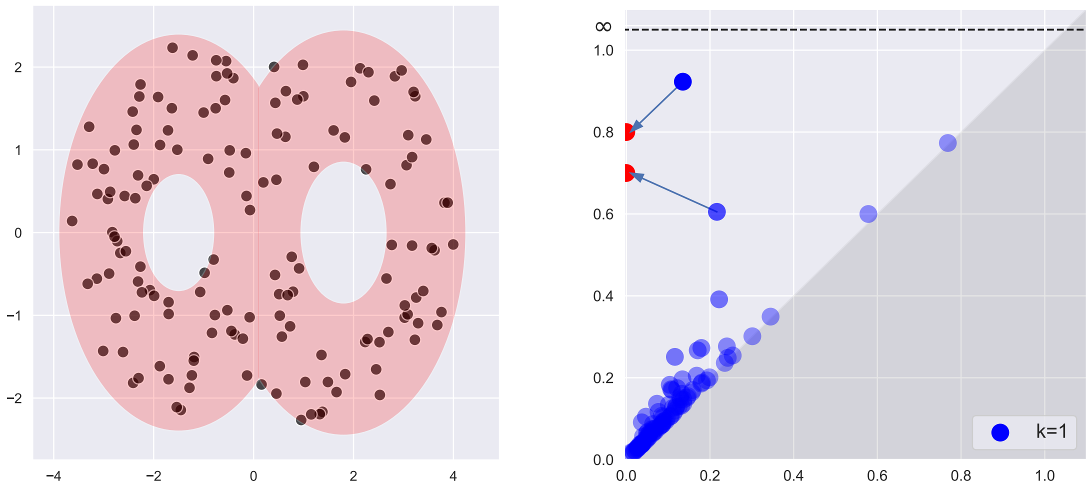

FIGURE 1. (Left) Points sampled uniformly from a horizontal figure eight (2 annuli) with radii $r = 0.7$ and $r = 0.8$ respectively. (Right) The corresponding persistence diagram (for the Čech complex). There are two blue points far from the diagonal corresponding to the holes in the figure eight. The red points and arrows show the corresponding classes in the underlying annuli.

More formally, we adopt the setting from manifold learning and assume the reader is familiar with persistent homology. Let $\mathcal{S} \subseteq \mathbb{R}^d$ be the support of a probability distribution, let $\mathbf{X} \subseteq \mathcal{S}$ be a random subset, and let K_r denote either the Čech or Rips complex generated by $\mathbf{X}$ at distance r. Note that while the vertex set of K_r is a subset of $\mathcal{S}$, there is no immediate way to map $\mathrm{H}_k(K_r)$ into $\mathrm{H}_k(\mathcal{S})$. Our first step is thus to define such a mapping that will be natural, in the sense that it preserves the intuitive meaning of inclusion. To do so, we will define a space $\mathcal{S}_r$, along with maps $i_*^r : \mathrm{H}_k(\mathcal{S}) \to \mathrm{H}_k(\mathcal{S}_r)$, and $j_*^r : \mathrm{H}_k(K_r) \to \mathrm{H}_k(\mathcal{S}_r)$. Mapping both $\mathrm{H}_k(\mathcal{S})$ and $\mathrm{H}_k(K_r)$ into a common space $\mathrm{H}_k(\mathcal{S}_r)$ we allow us find k-cycles in K_r that have a corresponding cycle in $\mathcal{S}$.

For the Čech complex, let $\mathcal{S}_r$ be the the r-offset of $\mathcal{S}$, i.e., all points in $\mathbb{R}^d$ which are at most distance r away from $\mathcal{S}$. The map i_*^r is induced by the inclusion $\mathcal{S} \hookrightarrow \mathcal{S}_r$. The map $j_*^r : \mathrm{H}_k(K_r) \to \mathrm{H}_k(\mathcal{S}_r)$ is induced by the inclusion $\mathbf{X}_r \hookrightarrow \mathcal{S}_r$, where $\mathbf{X}_r$ is the r-offset of $\mathbf{X}$, combined with the Nerve Lemma [12]. For the Rips complex, we take $\mathcal{S}_r$ to be the (infinite) Rips complex constructed over all points in $\mathcal{S}$, at distance r. From [30], it is known that for small enough ϵ, $\mathcal{S}$ and $\mathcal{S}_\epsilon$ are homotopy equivalent. The map i_*^r is then induced by the inclusion $\mathcal{S}_\epsilon \hookrightarrow \mathcal{S}_r$, combined with the homotopy equivalence $\mathcal{S} \simeq \mathcal{S}_\epsilon$. The map j_*^r is induced by the inclusion $K_r \hookrightarrow \mathcal{S}_r$.

We can now define the signal as follows. For any point $p \in \mathrm{dgm}_k$, let $\mathrm{b} = \mathrm{birth}(p)$, and let $[p] \in \mathrm{H}_k(K_\mathrm{b})$ be a representative of the homology class of p at its birth time. Note that we do not assume that $[p]$ is unique. We define the *signal diagram* as

$$\mathrm{dgm}_k^{\mathbf{S}} = \{p \in \mathrm{dgm}_k : j_*^{\mathrm{b}}([p]) \in \mathrm{Im}(i_*^{\mathrm{b}})\}.$$

In other words, p will be considered as a signal point if it matches a non-trivial cycle in $\mathcal{S}$ (via i_*^{b} and j_*^{b}). The *noise diagram* can be then defined as the remaining points

$$\mathrm{dgm}_k^{\mathbf{N}} = \mathrm{dgm}_k \setminus \mathrm{dgm}_k^{\mathbf{S}}.$$

Note that additional assumptions are needed if one wants to prove that the signal diagram captures any or all of the homology of $\mathcal{S}$. This has been the topic of numerous studies, from a geometric perspective [16, 20, 49] and from a probabilistic perspective [11, 26].

Remark 1. *The definition we provide here for the signal diagram, assumes that the signal is represented by the support of the probability distribution. In other words, we assume that we are able to sample directly from $\mathcal{S}$. This, however, ignores the plausible case, where the sample $\mathbf{X}$ is generated* around $\mathcal{S}$, *rather than*

directly on it. In such cases, we may consider the signal as coming from a super-level set of the density function (i.e., a sufficiently dense region), rather than the support, which might even be the entire $\mathbb{R}^d$. Thus, the precise definition of signal and noise becomes much more complex, and requires additional assumptions and techniques. For example, in [9, 39] *it was shown that the signal can be recovered by estimating the density function, and then taking the super-level sets of the estimates. While this is an interesting direction of research within the field, we leave it for future work.*

3. Complete experimental results

3.1. **Sampling from iid distributions.** We first examine the experimental results supporting Conjecture-1 more closely. To this end we examined the settings detailed in Tables 1 and 2. The π-values distributions for the Čech filtration are presented in Figure 4 and for the Rips filtration in Figure 5. We note that not all settings in Tables 1 and 2 were computed for both the Čech and the Rips complex. The reasons for that are purely computational. Some of the settings required more memory or computing power than we have at our disposal. Tables 3-4 provide the full list of iid-configurations tested, including the sample size used. The varying sample sizes are due to both our wish to explore the behavior at different values of n, and the computational limitation we have for computing the persistence diagrams. In Figures 6-9 we present the distribution of the ℓ-values for all the iid configurations tested.

space ($\mathcal{S}$)	intrinsic dimension (d)	embedding dimension
Box	2,3,4,5	d
Ball	2,3,4,5	d
Sphere	2,3,4	$d+1$
Torus	2	3
Henneberg surface	2	3
Neptune mesh	2	3
Klein bottle	2	4
Projective plane	2	4

Table 1. An exhaustive list of all iid uniform distributions tested. In the Čech complex, we computed all relevant dimensions of homology, $k = 1, \dots, d-1$. For computational reasons, we limited the Rips to $k = 1, 2$.

distribution ($\mathbb{P}$)	density function	space ($\mathcal{S}$)	dimension (d)
Beta(3,1)	x^2	$[0,1]^d$	2,3,4,5
Beta(5,2)	$x(1-x)^4$	$[0,1]^d$	2,3,4,5
Normal	$\exp(-x^2/2)$	$\mathbb{R}^d$	2,3,4,5
Cauchy	$(1+x^2)^{-1}$	$\mathbb{R}^d$	2,3,4,5

Table 2. An exhaustive list of all iid non-uniform distributions tested. The homology dimensions computed (k) are the same as in Table 1. The second column contains the expression of the 1-dimensional density used (up to a normalizing constant).

For details on the iid distributions used, see the Methods section in the paper body, however we include examples of the less standard point clouds in Figure 2. An example for the Vietoris-Rips and Čech complexes is presented in Figure 3.

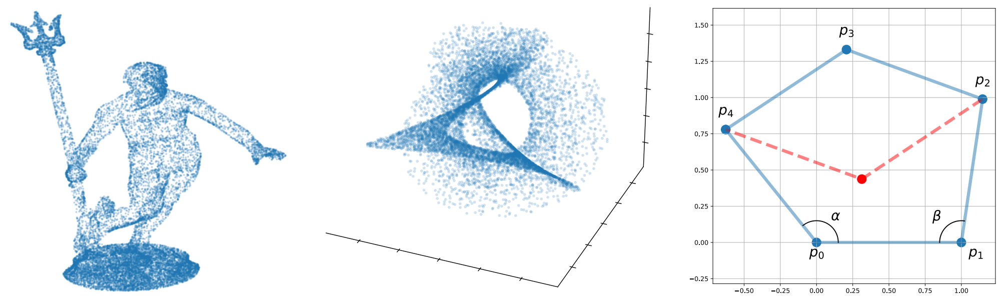

Figure 2. (left) a uniform sample of the Neptune statue. (middle) a sampling of the Hennenberg surface. (right) A example of a linkage configuration. The light colored vertex is the reflected option mentioned in our description.

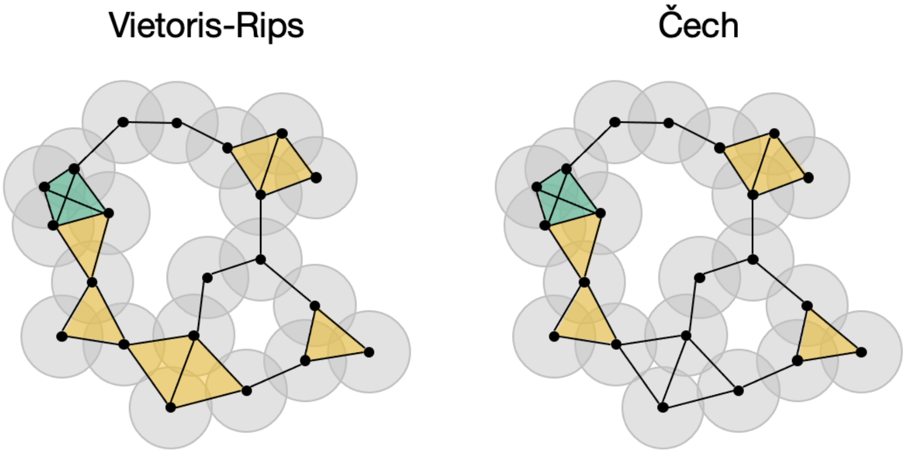

Figure 3. Examples for the Vietoris-Rips and Čech complexes, generated by the same point-cloud. Note that the two triangles at the bottom are present in the Rips complex (since all edges are included), but are missing from the Čech complex (since the balls do not have a common intersection).

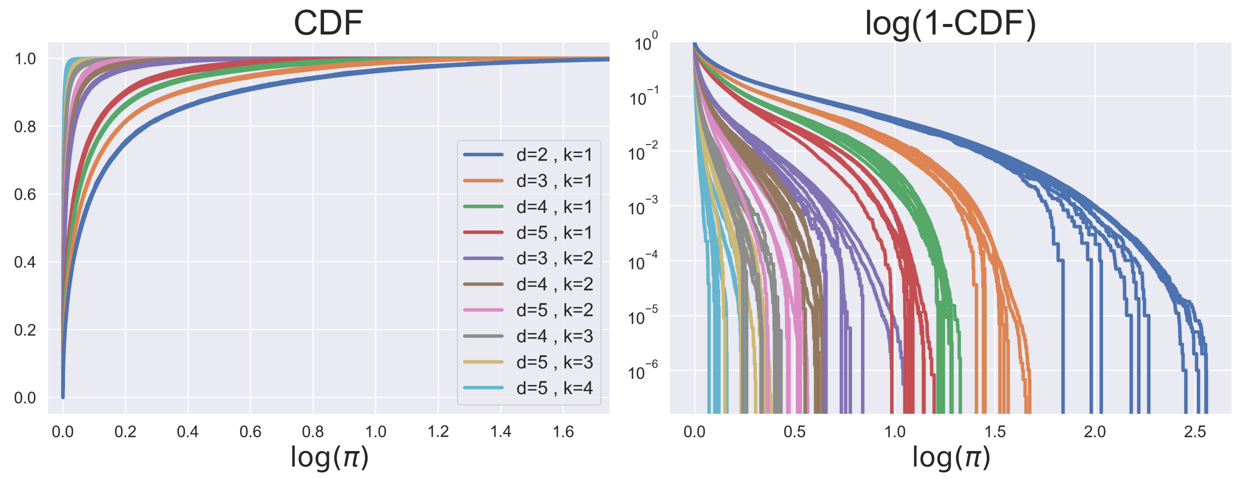

FIGURE 4. CDFs π-values in all iid settings tested, using the Čech complex. The complete list of models included in this figure is in Table 3

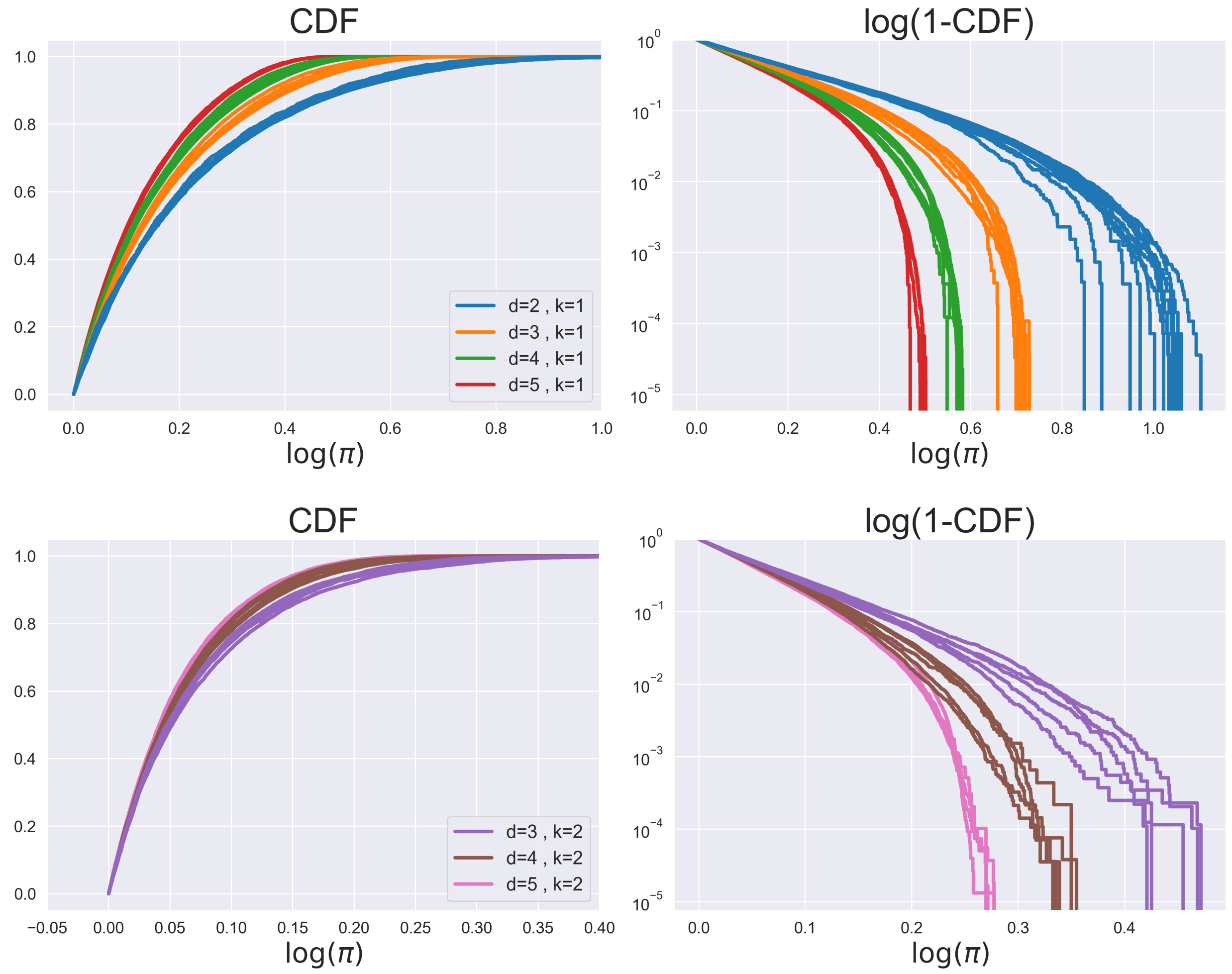

FIGURE 5. CDFs π-values in all iid settings tested, using the Rips complex. The complete list of models included in this figure is in Table 4

distribution ($\mathbb{P}$)	dimension (d)	homology degree (k)	sample size (n)
Box	2	1	1,000,000
Box	3	1-2	1,000,000
Box	4	1-3	50,000
Box	5	1-4	100,000
Ball	2	1	50,000
Ball	3	1-2	50,000
Ball	4	1-3	50,000
Ball	5	1-4	50,000
Annulus	2	1	50,000
Annulus	3	1-2	10,000
Annulus	4	1-3	10,000
Annulus	5	1-4	10,000
Sphere	2	1	100,000
Sphere	3	1-2	50,000
Sphere	4	1-3	50,000
Sphere	5	1-4	10,000
Beta(3,1)	2	1	50,000
Beta(3,1)	3	1-2	50,000
Beta(3,1)	4	1-3	50,000
Beta(3,1)	5	1-4	10,000
Beta(5,2)	2	1	100,000
Beta(5,2)	3	1-2	50,000
Beta(5,2)	4	1-3	50,000
Beta(5,2)	5	1-4	10,000
Cauchy	2	1	100,000
Cauchy	3	1-2	100,000
Cauchy	4	1-3	100,000
Cauchy	5	1-4	10,000
Normal	2	1	1,000,000
Normal	3	1-2	1,000,000
Normal	4	1-3	50,000
Normal	5	1-4	50,000
Torus	2	1	1,000,000
Klein	2	1	1,000,000
Projective	2	1	50,000

TABLE 3. Complete experimental list for the iid settings used with the Čech complex.

distribution ($\mathbb{P}$)	dimension (d)	homology degree (k)	sample size (n)
Box	2	1	100,000
Box	3	1-2	50,000
Box	4	1-2	50,000
Box	5	1-2	50,000
Ball	2	1	50,000
Ball	3	1-2	50,000
Ball	4	1-2	50,000
Ball	5	1-2	50,000
Annulus	2	1	10,000
Annulus	3	1-2	50,000
Annulus	4	1-2	10,000
Annulus	5	1-2	10,000
Sphere	2	1	50,000
Sphere	3	1-2	50,000
Sphere	4	1-3	100,000
Sphere	5	1-4	50,000
Beta(3,1)	2	1	50,000
Beta(3,1)	3	1-2	50,000
Beta(3,1)	4	1-2	50,000
Beta(3,1)	5	1-2	10,000
Beta(5,2)	2	1	50,000
Beta(5,2)	3	1-2	50,000
Beta(5,2)	4	1-2	50,000
Beta(5,2)	5	1	10,000
Cauchy	2	1	10,000
Cauchy	3	1-2	50,000
Cauchy	4	1-2	50,000
Cauchy	5	1-2	10,000
Normal	2	1	50,000
Normal	3	1-2	50,000
Normal	4	1-2	50,000
Normal	5	1-2	50,000
Torus	2	1	1,000,000
Klein	2	1	10,000
Projective	2	1	50,000
Linkage	2	1	50,000
Neptune	2	1	50,000

TABLE 4. Complete experimental list for the iid settings used with the Rips complex.

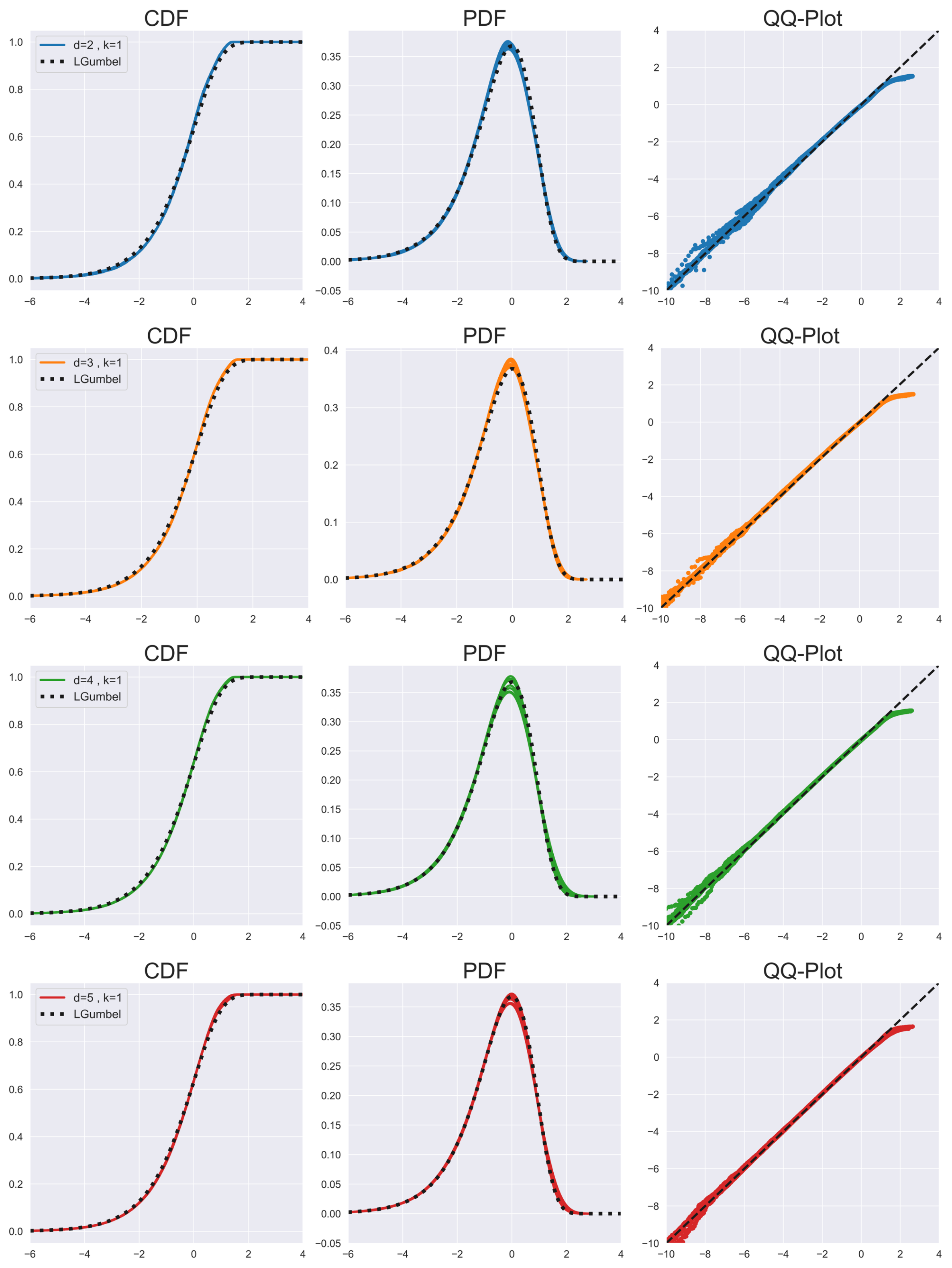

FIGURE 6. The ℓ-values distribution for the Čech complex – part 1/3.

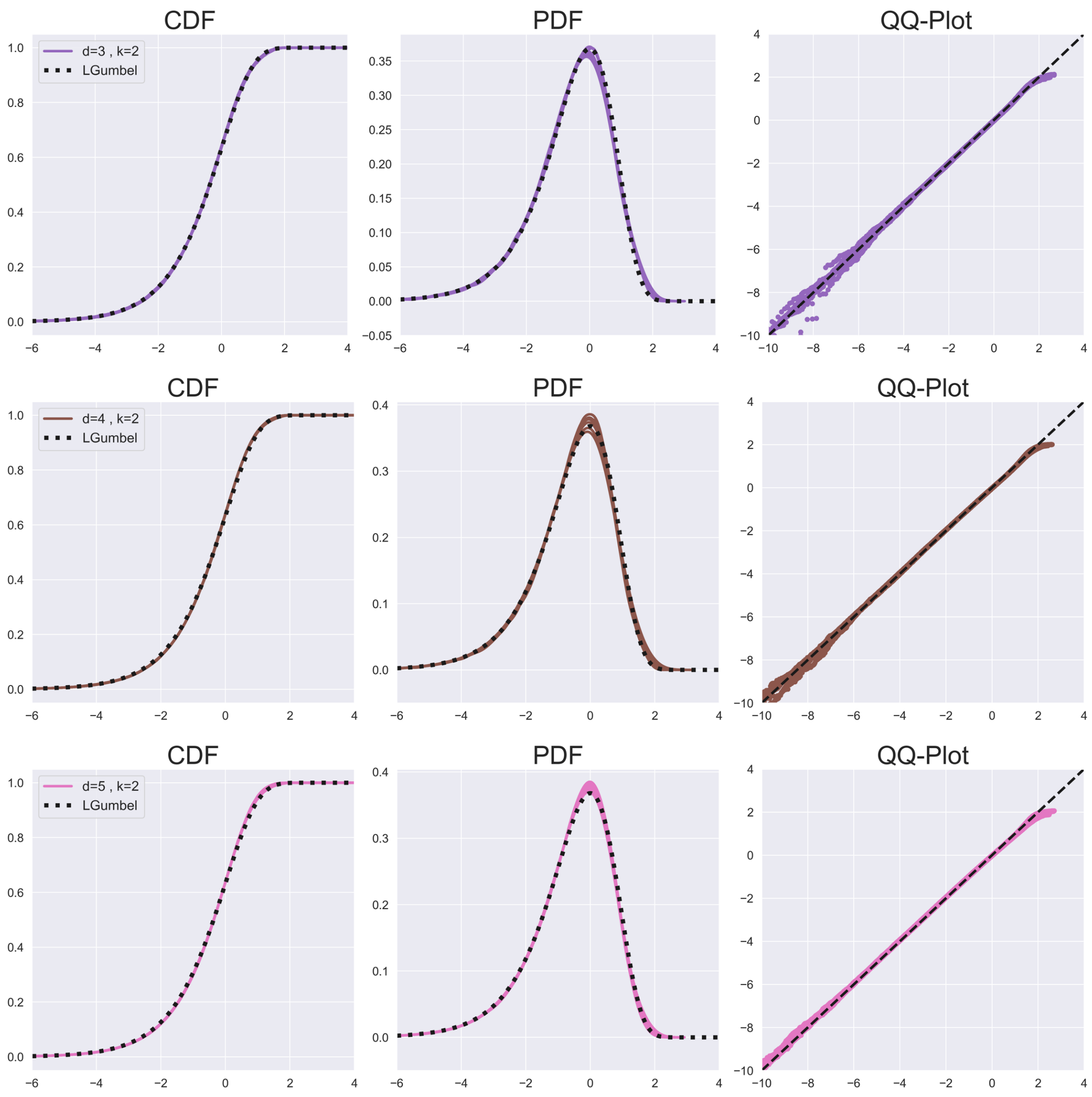

FIGURE 7. The ℓ-values distribution for the Čech complex – part 2/3.

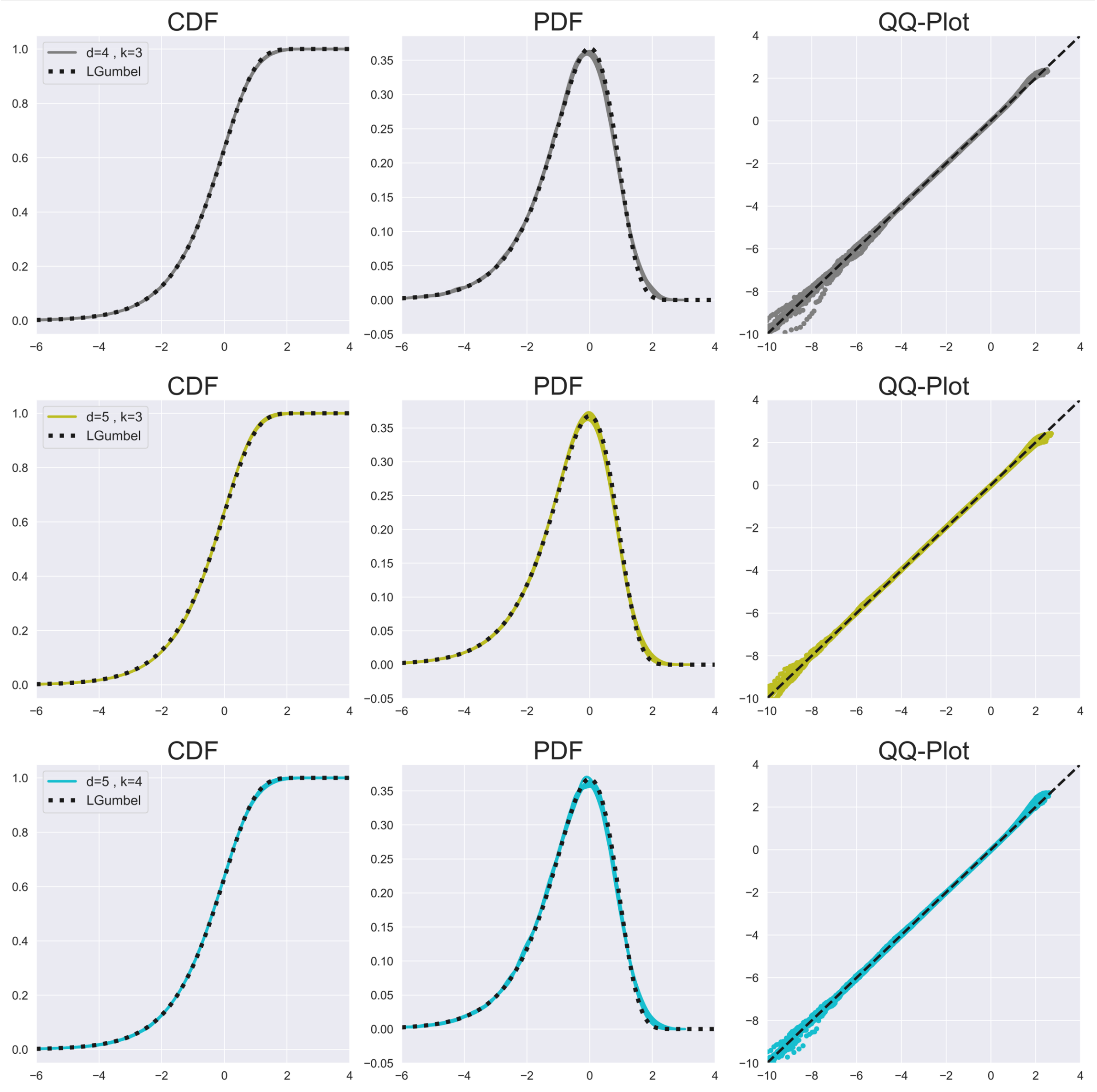

Figure 8. The ℓ-values distribution for the Čech complex – part 3/3.

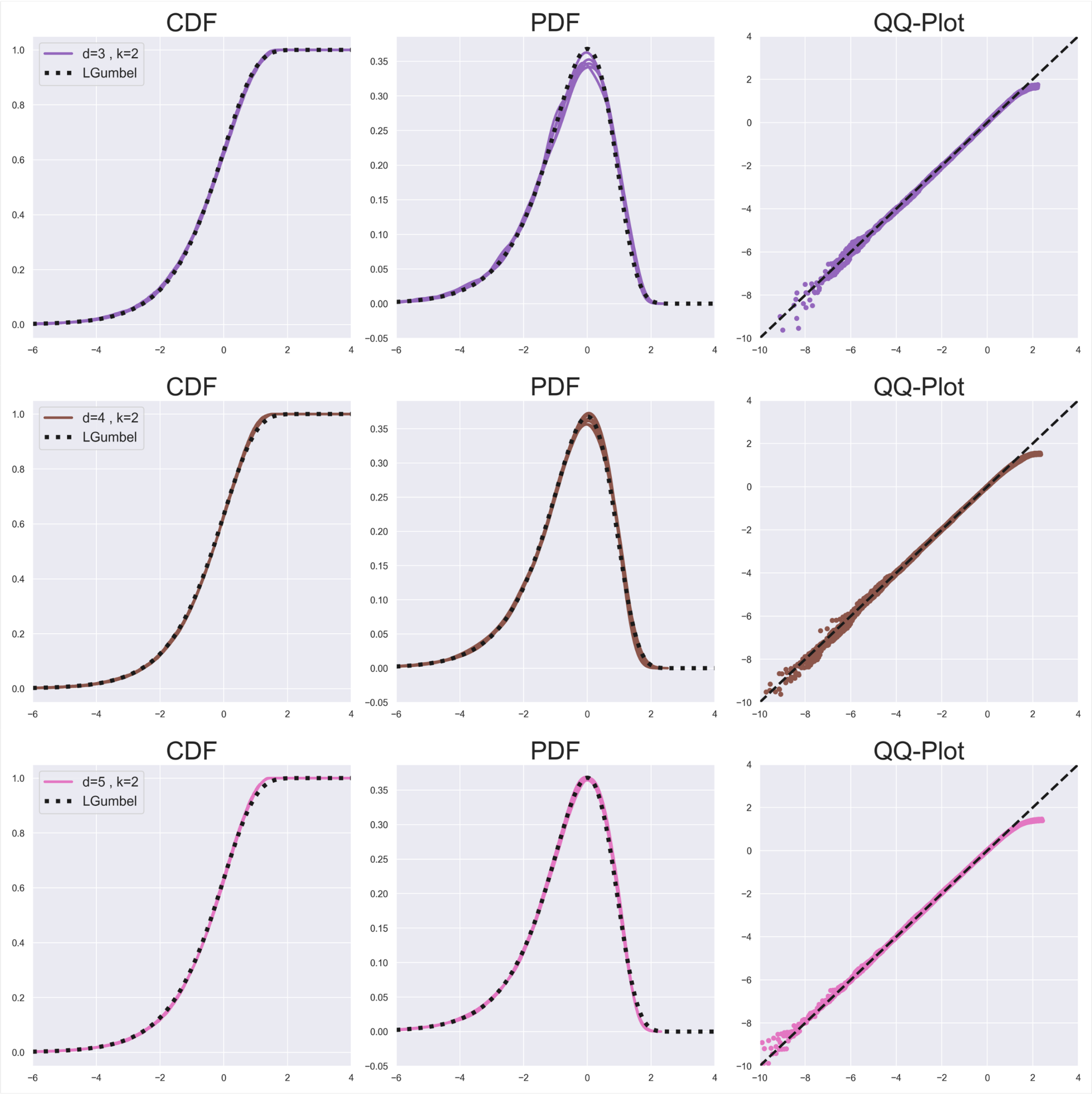

FIGURE 9. The ℓ-values distribution for the Rips complex – part 2/2.

3.2. **Sampling from non-iid distributions.** As mentioned in the paper, we tested two examples of non-iid point-clouds – a d-dimensional Brownian motion and a Lorenz dynamical system (see the Methods section for details on how these were generated), see Figure 10 for examples of these point clouds.

The results for Brownian motion can be seen in Figures 11-12. In the top two rows of Figures 11-12, we present the distribution of π-values for Brownian point-clouds (in color) compared to a selected baseline from the iid distributions (in gray). We included these figures here to show that the Brownian samples behave very differently from the iid samples. Nevertheless, the bottom two row presents the distribution of the ℓ-values, which seems to follow the same LGumbel distribution as the iid case. All samples here are of size $n = 50,000$. Similarly for the Lorenz dynamical system, the results are shown in Figure 13. The sample size for the Čech is $n = 100,000$, and for the Rips is $n = 50,000$.

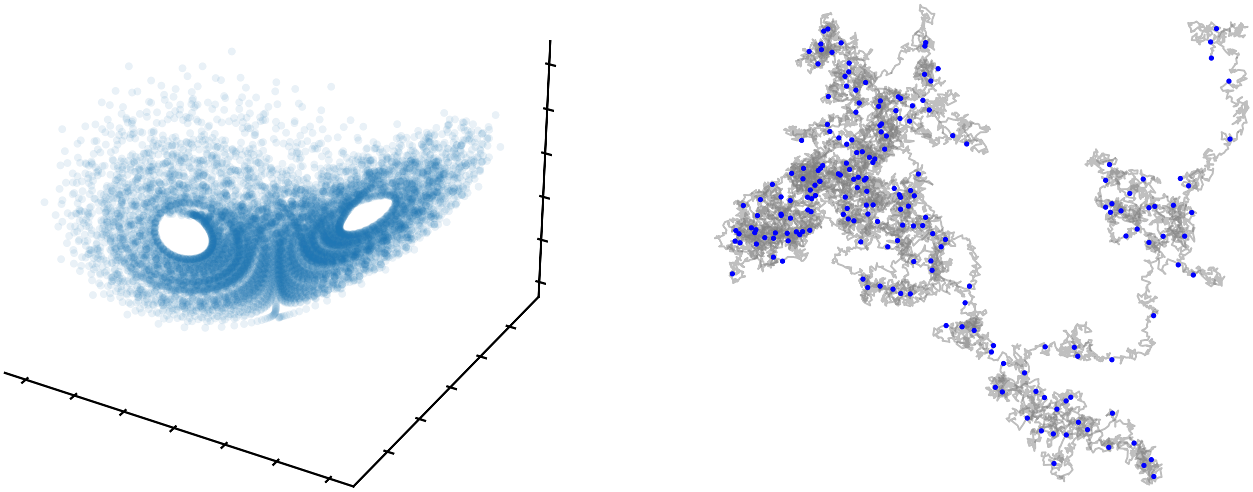

Figure 10. Two examples of non-iid sampled point clouds we tested. (left) A sample of the Lorenz system, a well-known chaotic system. (right) A sample of Brownian motion in the plane.

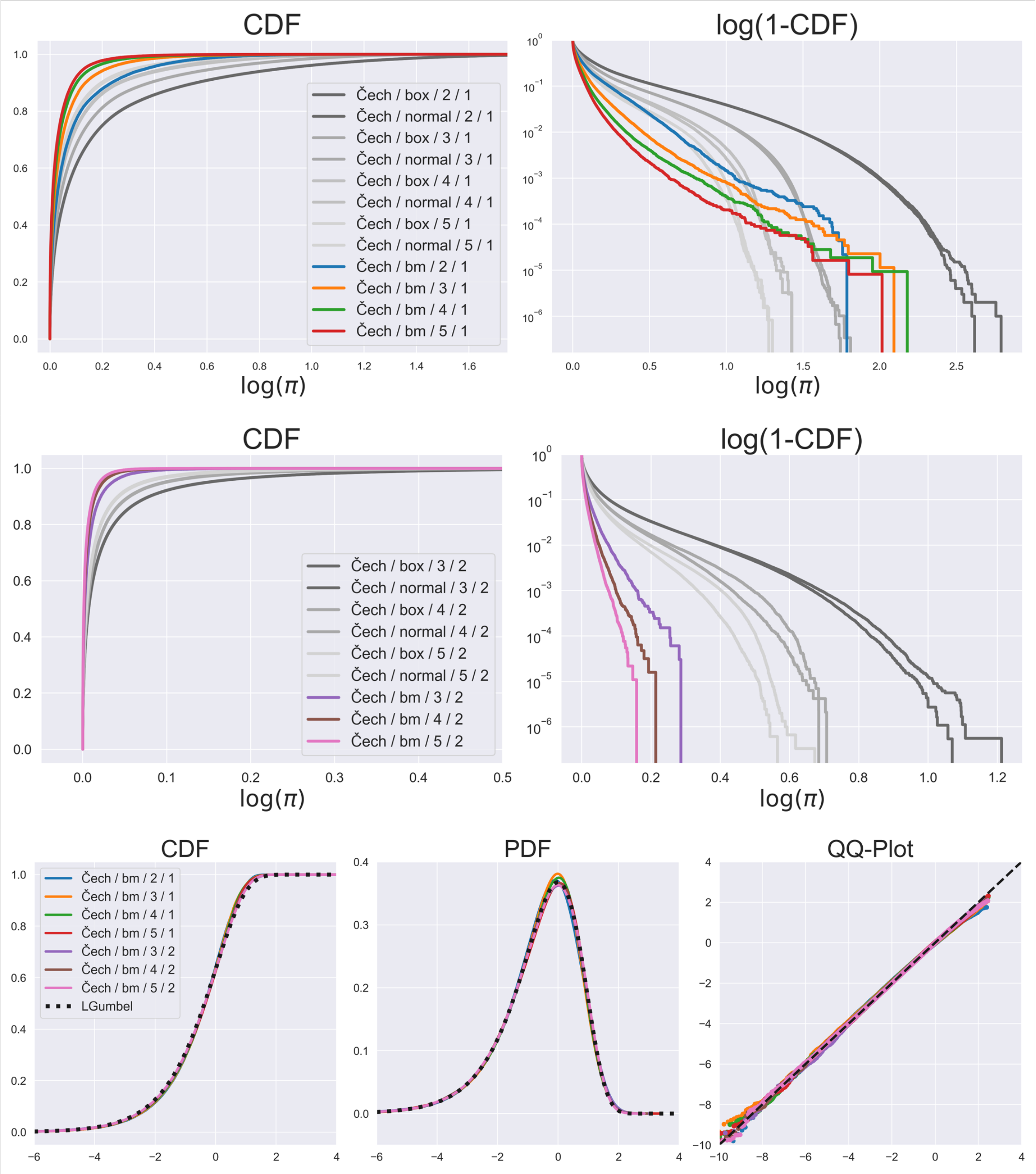

FIGURE 11. Sampling from a Brownian motion, the Čech complex.

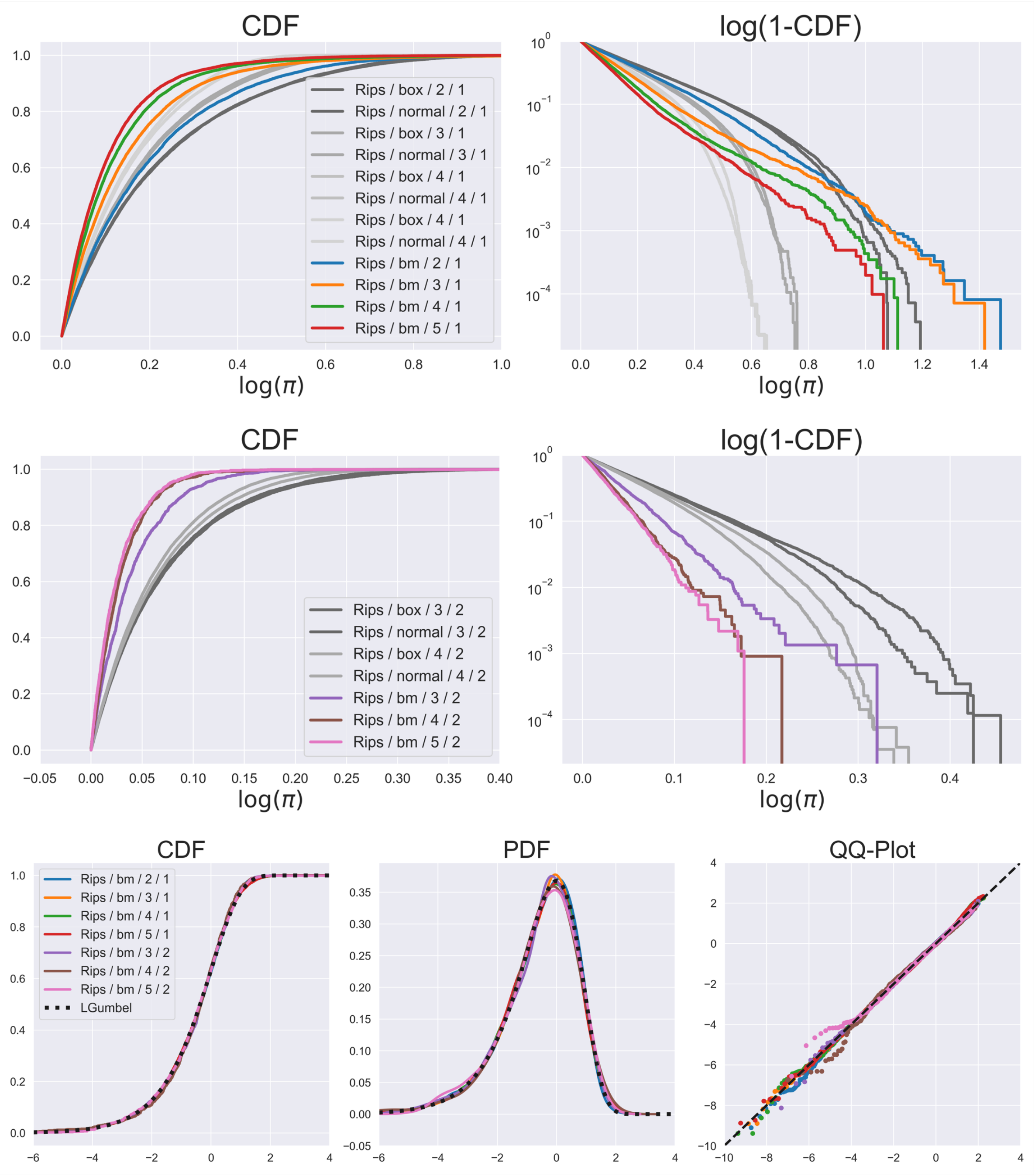

Figure 12. Sampling from a Brownian motion, the Rips complex.

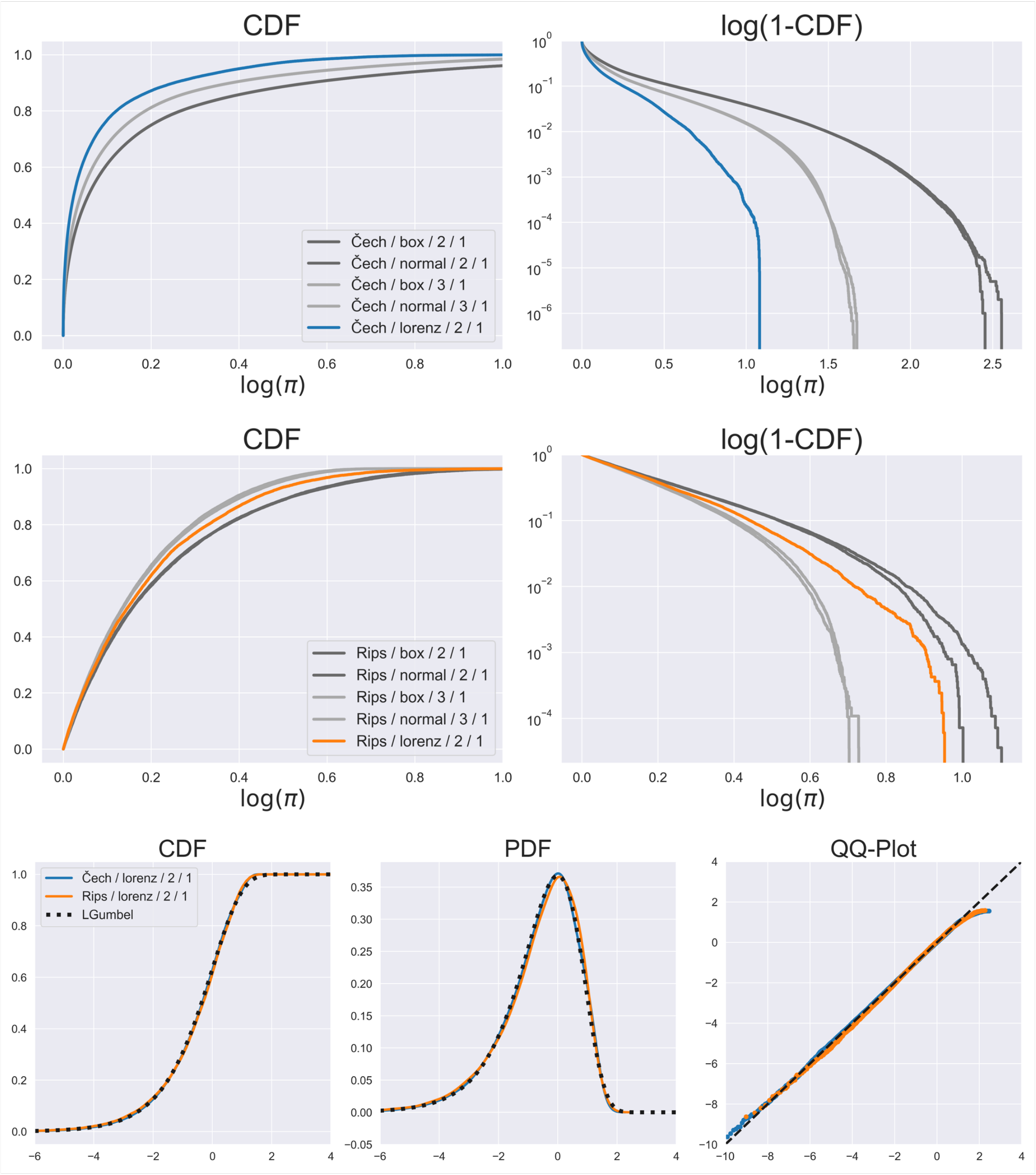

FIGURE 13. Sampling the Lorenz system – both Čech and Rips.

3.3. **Testing on real data.** As described in the paper, we also tested the strong-universality conjectures against three examples of real data - patches of natural images, sliding windows of voice recordings, and sentence embeddings.

The results for the Image Patches dataset are presented in Figure 14. In addition to taking the original 8-dimensional point-cloud, we also examined its lower dimensional projections for dimensions $d = 3, \dots, 7$. The sample size used was $n = 50,000$ for all dimensions. Likewise the results for the Takens' embedding of a voice recording are presented in Figure 15. The results for the sentence embeddings is in Figure 16. The Bible has 29,812 sentences, while Moby Dick has 8,762 and Ulysses has 23,641.

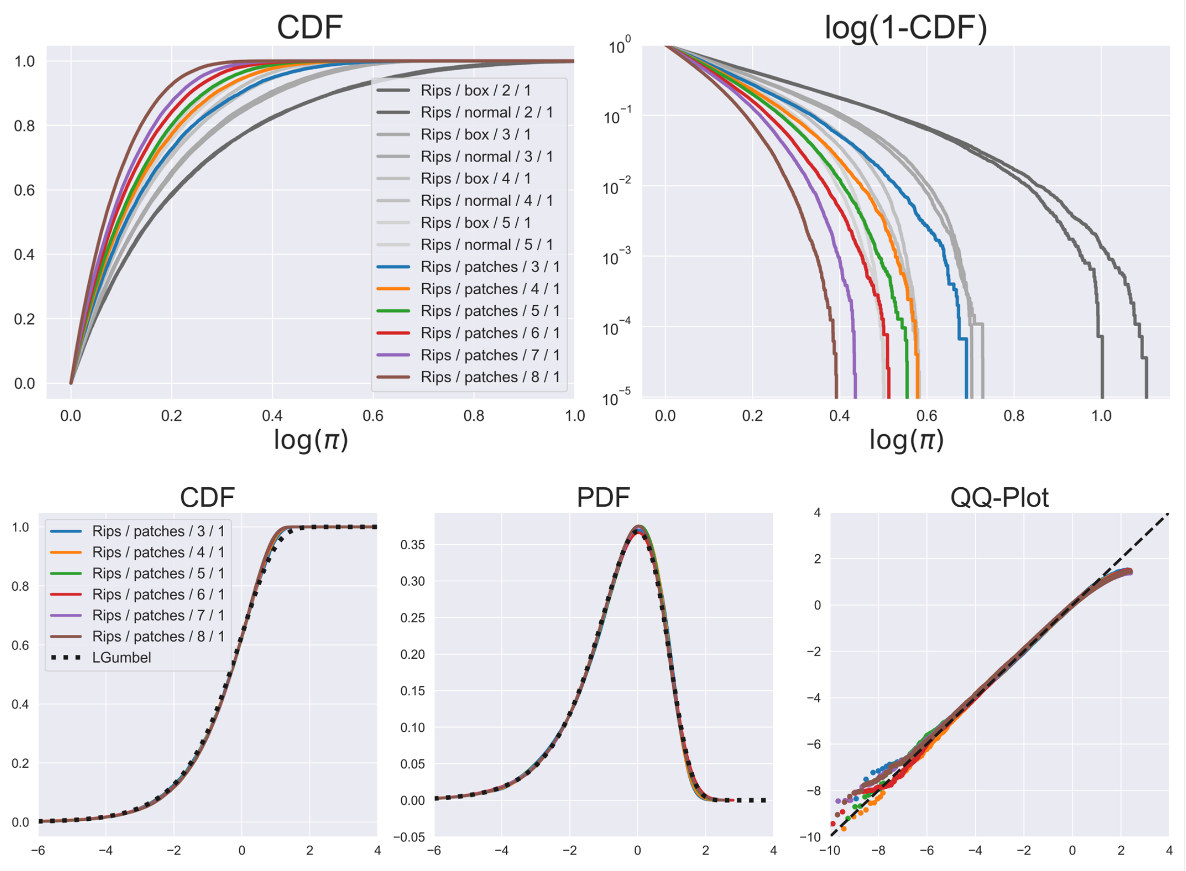

FIGURE 14. Sampling patches of natural image, Rips complex.

3.4. **Goodness of fit.** As stated briefly in the paper, we wanted to perform a goodness-of-fit test, to provide further evidence for the validity of our conjectures. To this end, we used the two-sided Kolmogorov-Smirnov (KS) test, with the null hypothesis that the distribution of the ℓ-values is LGumbel. We tested all the 210 different settings presented in the previous sections, and for none of them the null hypothesis was rejected (with $\alpha = 0.05$). Furthermore, it can be observed in Figure 17 that the KS statistic values are concentrated away from the critical value. This is a very positive reinforcement to our conjectures. A few comments are in place here:

- The accurate conclusion from this KS test, is *not* that the ℓ-values have an LGumbel distribution, but rather that our experimental results do not contain sufficient evidence to the contrary. This is, however, the best case scenario we can hope for in such goodness-of-fit testing.
- As we mentioned in the second remark following Conjecture 3, our results demonstrate some deviation of the tails which is a consequence of slow convergence rates. The deviation of the tail interferes with the performance of the KS test, especially for large sample sizes. Therefore, we ran the test on

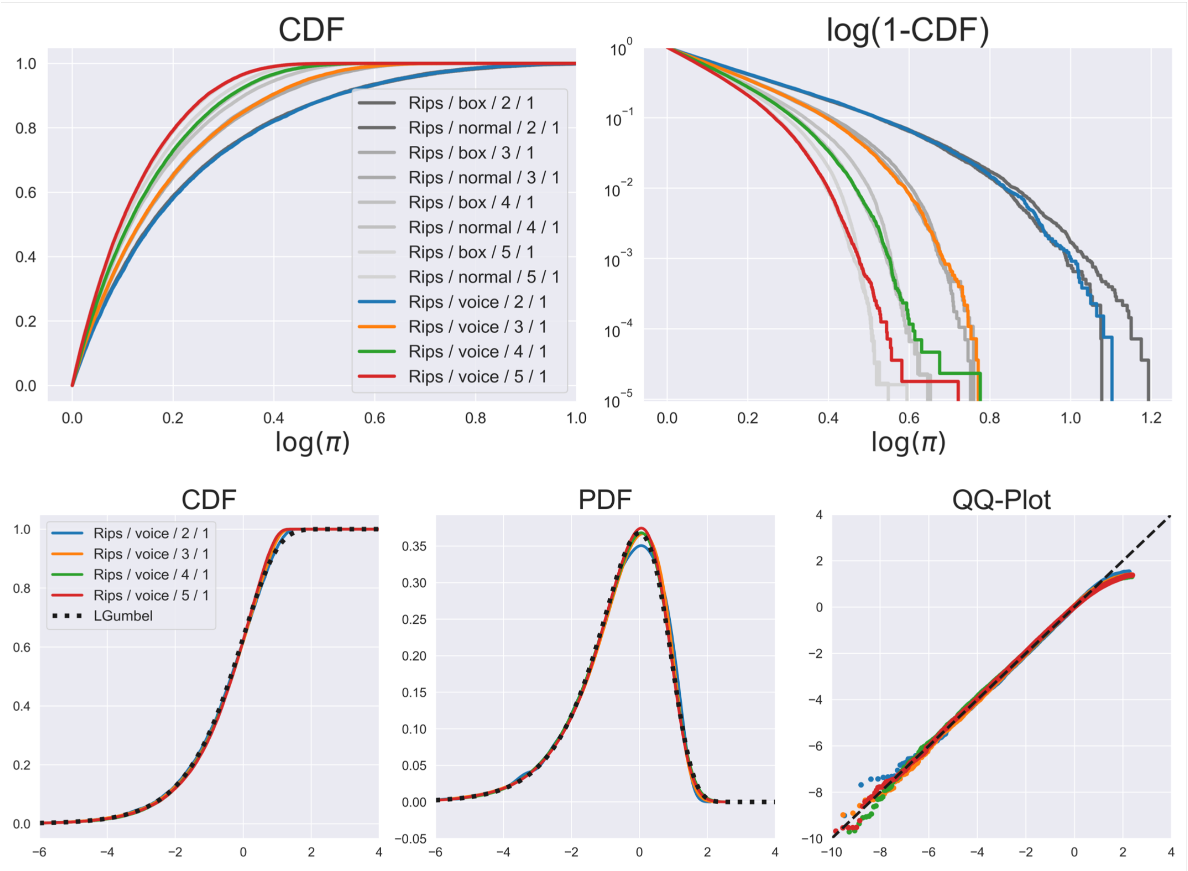

FIGURE 15. Sampling sliding windows from a voice recording, Rips complex.

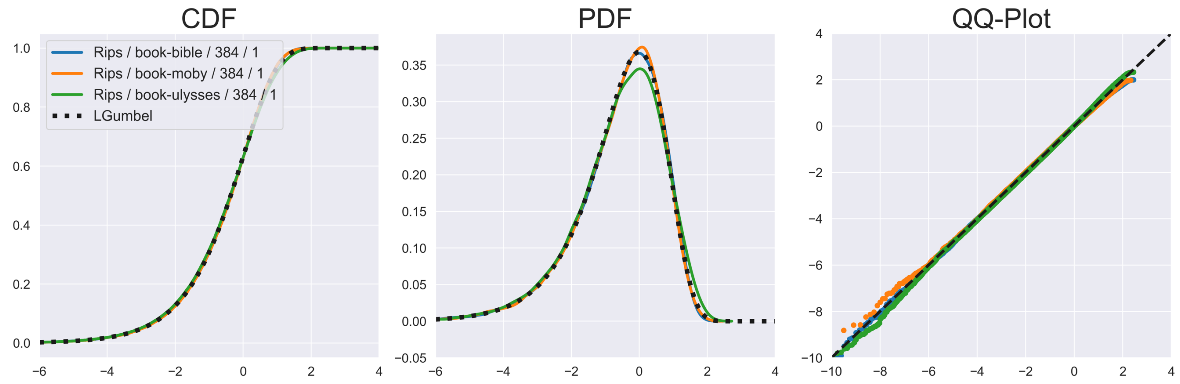

FIGURE 16. Sentence embedding from different books, Rips complex. As the dimension is very high (384), we do not show the comparison with the other distributions.

a small ($n = 1,000$) random sub-sample of the persistence values. This also ensures that we have almost-iid samples of the distribution (see Section 4), which is a requirement of the KS-test. We should note that the results presented are quite robust, in the sense that in repeated trials (with different sub-samples) we received similar results.

- As we are running 210 tests simultaneously, this should be considered as a case of multiple-hypothesis testing. The p-values were therefore adjusted using the Bonferroni correction.

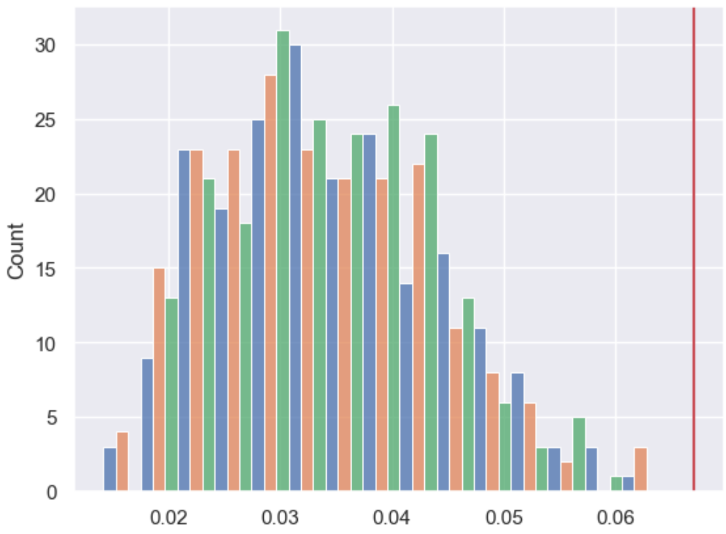

FIGURE 17. Kolmogorov-Smirnov statistics. We present the histogram for the KS statistic values for the entire collection of 210 point-clouds, testing each point-cloud against the Gumbel distribution. We present three independent instances, where in each instance we generated a random sub-sample of $n = 1000$ ℓ-values to perform the KS-test on. Note that the histogram seems to be robust, and concentrated away from the critical value (for $\alpha = 0.05$), represented by the vertical red line.

3.5. **Non-universal examples.** Finally, we present three examples of point-clouds which do not follow the universal behavior described in the paper. They have properties which do not allow for points to be "too close" to each other or have a large number of significant cycles. We first describe the models, and the results can be seen in Figure 18 along with examples of the point clouds in Figure 19.

(1) **Ginibre Ensemble**: Our first example is the Ginibre ensemble [28]. This is a random point cloud of size n in two dimensions (in the complex plane), where the coordinates of the points are the eigenvalues of an $n \times n$ matrix with entries that are i.i.d. uniform random complex values. In the experiment below we chose $n = 10,000$.
(2) **Perturbed Grid**: Our second example is a perturbed grid in two dimensions. First taking points on a grid on the square, we perturb the points uniformly at random at 20% of the spacing. The example we show was on a 150×150 grid, so $n = 22,500$.
(3) **Bubbles**: Our last example is where the number and sizes of empty regions was maximized using gradient descent or flow on point [53], resulting in a "bubble" like structure on a two dimensional torus. We then added a small amount of noise to verify that the point cloud still did not exhibit universal behavior. In the result, n was $10,000$.

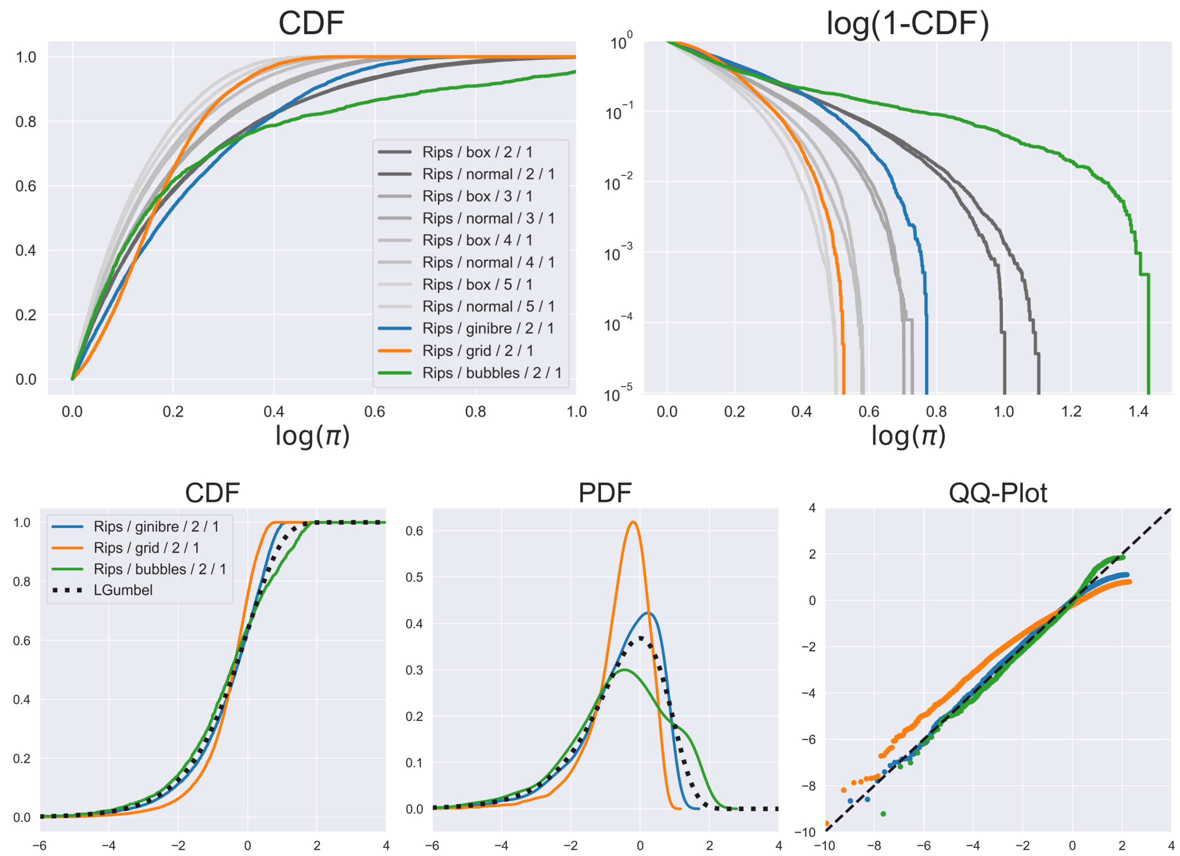

Figure 18. Persistence distribution for point-clouds which do not satisfy universality, Rips complex.

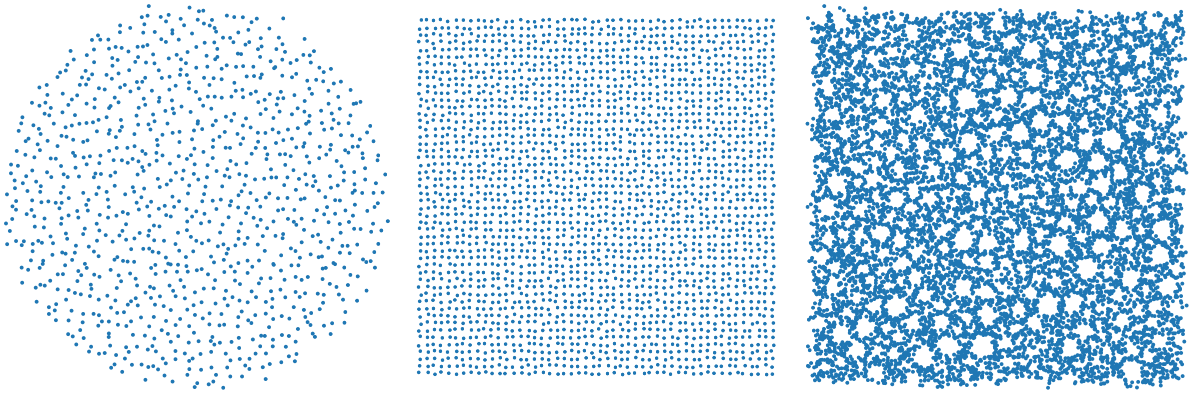

Figure 19. Examples of point-clouds which do not satisfy universality. (left) 1,000 point sample from the Ginibre ensemble, (middle) A 50×50 perturbed grid, (right) The perturbed bubbles structure with 10,000 points.

4. Dependency testing

In the paper, our first two conjectures, i.e. Conjectures 1 and 2, are concerned with the limit for the empirical measure of persistence π-values and ℓ-values, respectively. The way we interpret these conjectures is as follows. Given a random sample $\mathbf{X}_n \sim \mathbb{P}_n$, the persistence diagram dgm_k is a random point process

in $\mathbb{R}^2$, i.e. a collection of random points $\{p_1, \ldots, p_M\} \subset \mathbb{R}^2$, where M itself is random. Note that there is no natural ordering to the points in a diagram, and to some extent their ordering is algorithm-specific. Therefore, in our point process model we will assume that the points are ordered completely at random. In practice, given the output of a persistent homology algorithm $\mathrm{dgm} = \{p_1, \ldots, p_m\}$, where $p_i = (\mathrm{b}_i, \mathrm{d}_i)$, we will consider the randomly ordered sequence $(p_{i_1}, \ldots, p_{i_m})$ where $(i_1, \ldots, i_m)$ is a random permutation of $(1, \ldots, m)$. In the following, we will always assume that $p_1, \ldots, p_m$ are already randomly ordered. We denote their persistence values by $\pi_i = \pi(p_i)$ and $\ell_i = \ell(p_i)$. Given the size of the diagram $M = m$, one can show that the random variables $\pi_1, \ldots, \pi_m$ all have the same marginal distribution (the same goes for $\ell_1, \ldots, \ell_m$). This distribution is approximated by the empirical measures we studied earlier (i.e., Π_n for π_i, and $\mathcal{L}_n$ for ℓ_i). However, even if we knew what the distribution of π_i is exactly, it would not tell us anything about the *joint m-dimensional distribution* of $(\pi_1, \ldots, \pi_m)$, and in particular about the dependency relationship between the different π-values (or ℓ-values)

To study the dependency between cycles within a single diagram, we conducted the following experiment. We generated N independent point-clouds, and computed their persistence diagrams, From each diagram we randomly sampled 25 points, denoted $p_1, \ldots, p_{25}$, and placed their corresponding ℓ-values in a vector of the form $L = (\ell_1, \ldots, \ell_{25}) \in \mathbb{R}^{25}$. We took $N = 100,000$ such realizations, from which we estimated both the covariance and the distance-covariance [51] matrices. As these experiments are quite costly (computing $100,000$ persistence diagrams for an individual setting), we only examined two cases here – the 2-dimensional box, and the 2-dimensional Brownian sample, in order to compare an iid setting with a non-iid one.

The results are presented in Figure 20. In addition to the results for the box (right), and the Brownian motion (middle), we also included the covariance and distance-covariance matrices, computed from 25 independent LGumbel variables. In addition to the matrices look rather similar, we also include the mean and maximum absolute values form these matrices in Table 5. We note that not only are these values quite low, they are comparable to the values obtained in an empirical covariance matrix obtained by generating iid random variables from the LGumbel distribution.

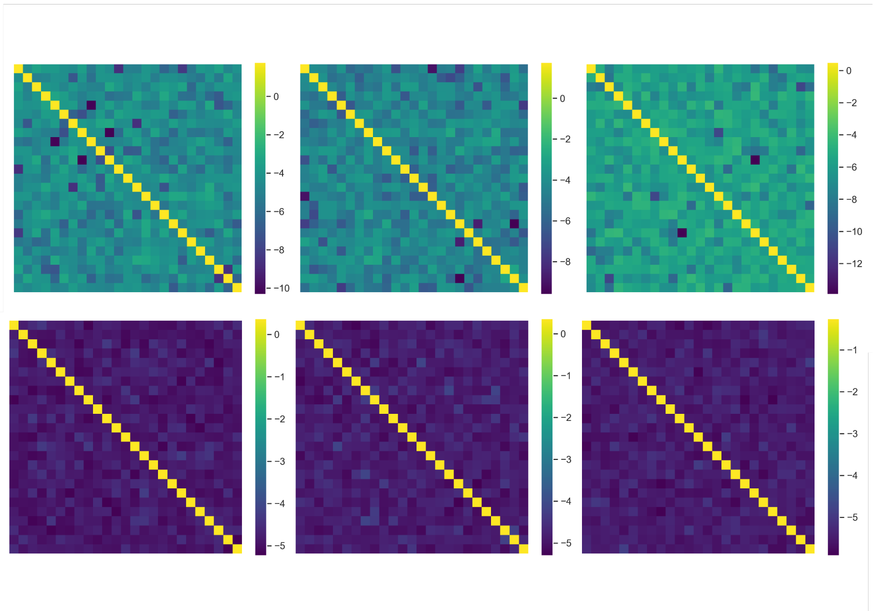

Figure 20. The covariance matrix (top) and distance covariance matrix (bottom), for $\ell_1, \ldots, \ell_{25}$, estimated from $N = 100,000$ repeated trials. (left) The ℓ-values are generated from the uniform distribution on the 2-dimensional box. (middle) The ℓ-values are generated by sampling the 2-dimensional Brownian motion. (right) The ℓ-values here are simply taken to be 25 iid random LGumbel variables.

	mean-corr	mean d-corr	max-corr	max-dcorr
2d-box	0.00233	0.00535	0.00887	0.01002
2d-brownian	0.00248	0.00540	0.01139	0.01128
iid-LGumbel	0.00242	0.00525	0.00843	0.00999

Table 5. Comparing correlations between ℓ-values in a box, Brownian motion, and iid LGumbel variables. We compute the mean and maximum absolute values for both the correlation matrix and the distance-correlation of the 25 ℓ-values extracted from the persistence diagrams.

5. Exploring the value of B

Returning to strong universality (Conjecture-2), notice that the only part that depends on the distribution generating the point-cloud is the normalization value $B = -\lambda - A\bar{L}$, where λ is the Euler-Mascheroni constant. Since $\bar{L}$ is the average of all $\log\log(\pi)$ values (a proxy for their mean value), it will depend on the distribution of the points, as we have seen in Section 3. It is therefore natural to ask about the relationship between B and the model parameters $(\mathbb{S}, \mathcal{T}, k)$.

In Figure 21 we explore the estimated values of B, showing the means (with error bars) versus the dimension, for different iid settings. As suggested by Conjecture-1, the value of B depends on $d, \mathcal{T}, k$, but is otherwise independent of $\mathbb{S}$. Figure 21 also suggests a linear relationship between B and the sampling dimension (d). A thorough investigation of this relationship remains a future work.

We also include experiments on stratified samples, which are iid but of mixed dimensions (between 2 and 3). For the dimension value (the x-axis), we use the weighted average corresponding to the percentage of points sampled from each stratum. Hence if 20% of the samples come from the 3 dimensional manifold and 80% from the 2 dimensional manifold, we assign a dimension of 2.8 to the sample. We can further see that the values are interpolating between dimension 2 and 3, but this is not a linear relationship. This could be due to the simple estimate of the dimension, as the remaining relationships seem quite linear. The precise relationship is difficult to ascertain due to the limited range of dimensions and we defer it to future work.

In Figures 22 and 23, we show the distributions of our estimates of B, across the various models, using sample size of $n = 50,000$. As can be seen in the figures, the Čech complex estimates are much more concentrated than the Rips ones, and the distributions are more spread out in higher homological dimensions. We believe that this is due to slower convergence in these cases (in terms of the number of points).

Finally, we wish to explore the connection between estimation variance and the sample size n. In the case of Rips complex, the variance of the estimates of B seems to be quite small in the compact-uniform case (e.g. on a box), compared to the variance emerging when sampling, for example, from the non-compact distribution (normal and Cauchy). In Figure 24, we fix $d = 3$ and $k = 1$, and present the distribution for the box compared to the non-compact cases. The sample-size for the box was fixed $n = 50,000$, and for the non-compact distribution we tested $n = 10k, 20k, 50k$, and $100k$. We observe that as n increases the distribution of the non-compact cases moves closer to the box case. This serves as an evidence that in the limit all cases have indeed the same value of B (supporting Conjecture-1), it is just that some cases converge slower than others.

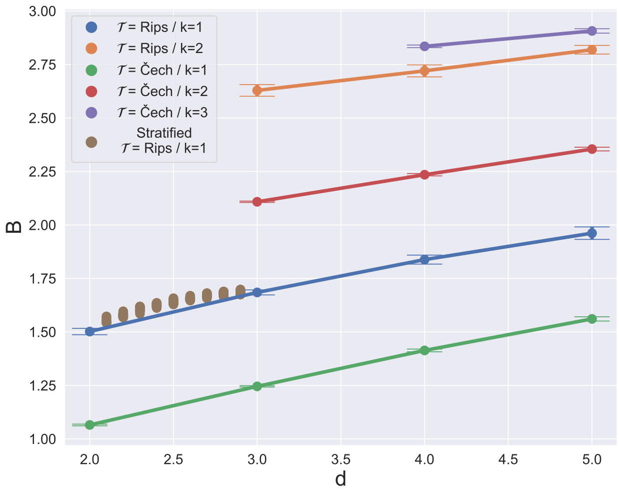

Figure 21. The estimated value for B, for iid samples with various choices of $(d, \mathcal{T}, k)$. We present the mean ±standard deviation of our estimates across various models. Here, we added stratified space samples, which are mixtures of different dimensional spaces (a plane embedded in a cube).

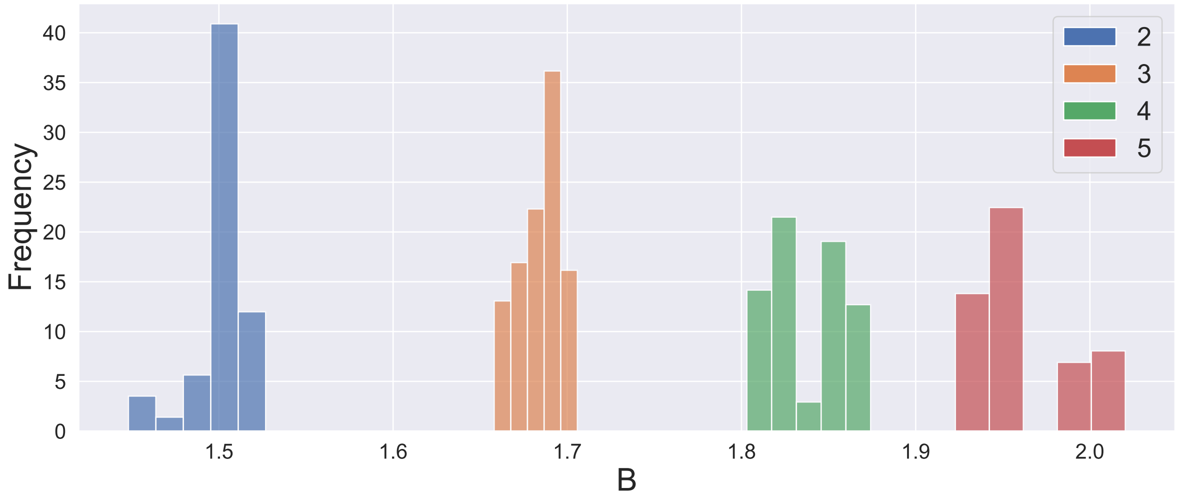

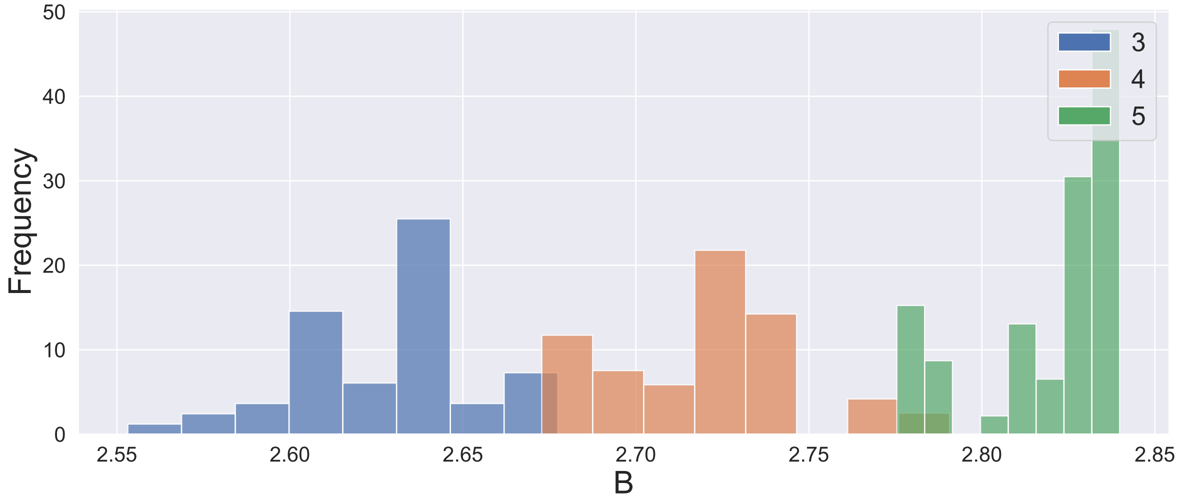

FIGURE 22. The distributions of the estimated B values, for the Rips complex, across different models for $k = 1$ (top) and $k = 2$ (bottom).

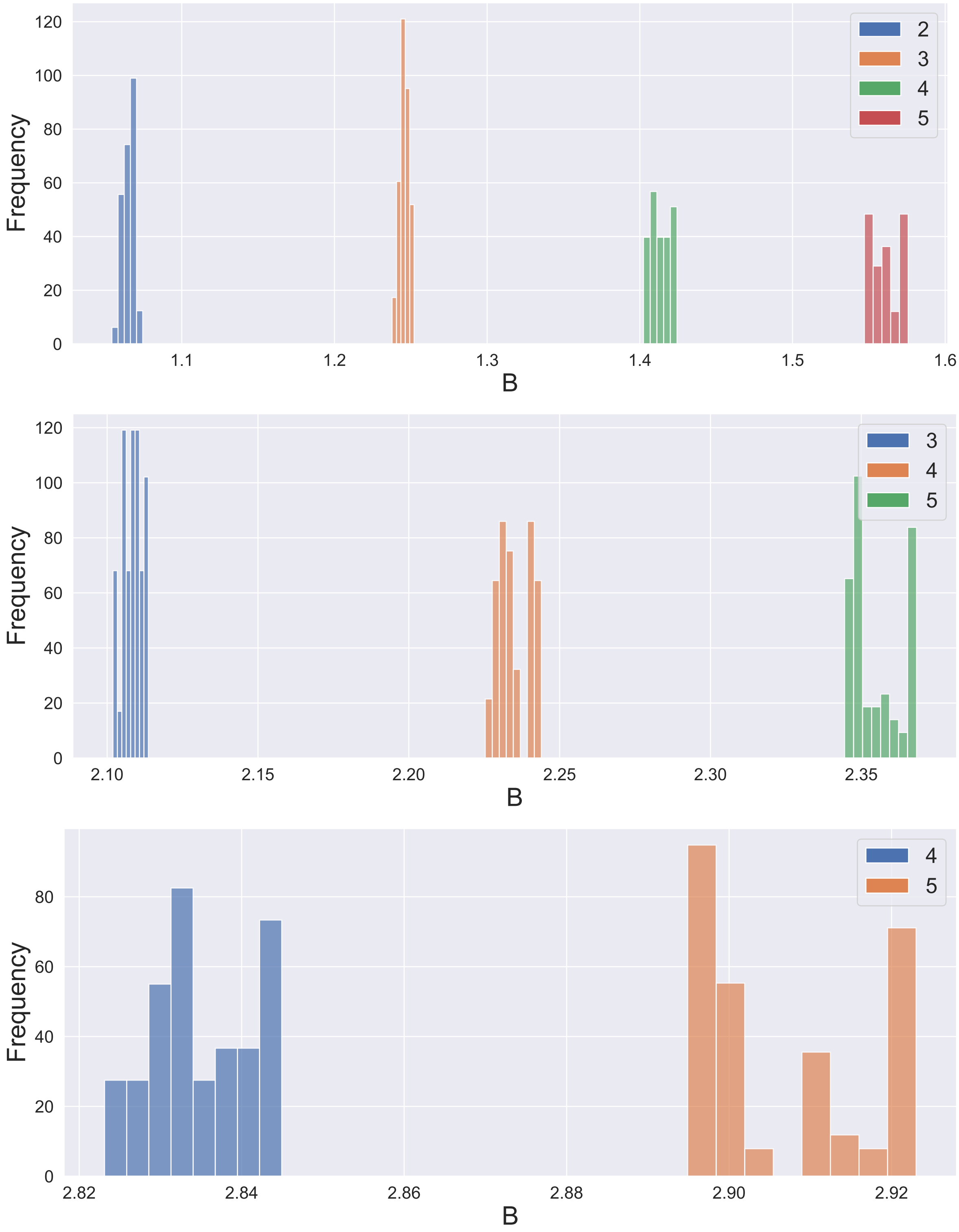

FIGURE 23. The distributions of the estimated B values, for the Čech complex, across different models for $k = 1$ (top), $k = 2$ (middle), and $k = 3$ (bottom).

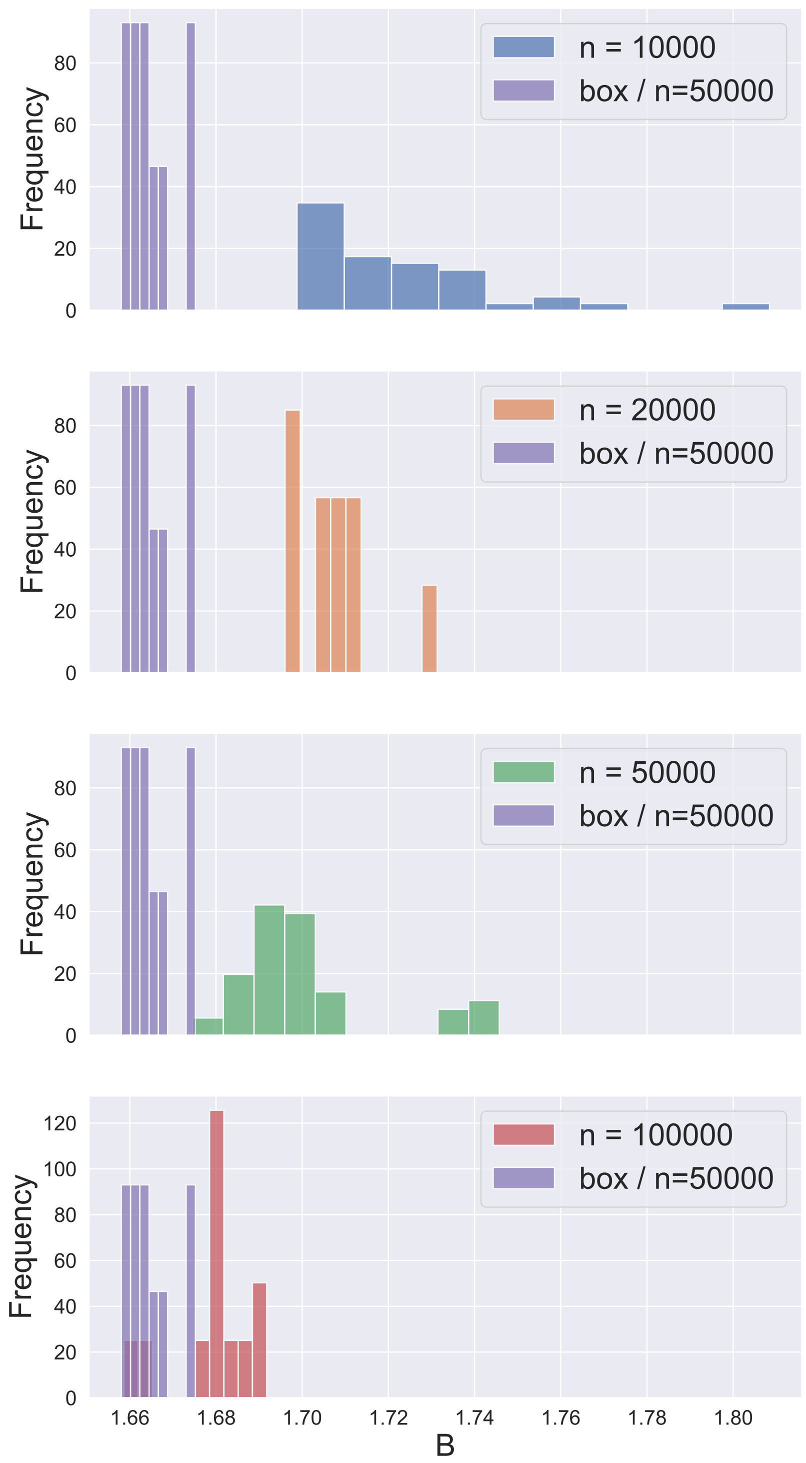

FIGURE 24. The distributions of the estimated value of B, for different values of n. We compare the non-compact distributions in $\mathbb{R}^3$ (normal, Cauchy) for $n = 10k, 20k, 50k, 100k$, with the uniform distribution on $[0,1]^3$, $n = 50k$ (in purple).

6. Hypothesis Testing Examples

In the paper we discussed our new hypothesis framework, and demonstrated it on the figure-8 example. In this section, we wish to present several more experiments testing this framework. In all our experiments, we set the desired significance level to be $\alpha = 0.05$.

Computing p-values. We start with a toy example to test our p-value computation. We sampled 1,000 points on an annulus in $\mathbb{R}^2$, whose outer radius is 1 and the inner radius is varied. In this case we expect to detect a single 1-cycle, corresponding to the hole of the annulus. For each value of r we computed the persistence diagram, and counted the number of cycles that are declared as significant (i.e., p-value $< \frac{\alpha}{|\,\mathrm{dgm}\,|}$). In Figure 25 on the right we present the results of this experiment with the curve showing the average number of significant cycles detected, over 1,000 repetitions, versus the inner radius of the annulus. In this figure we also show the persistence diagrams (left) of two realizations with different radii – one where the cycle is detected as significant, and one where it is not. For each diagram, the line of significance (corresponding to $\alpha = 0.05$) is shown (the dotted and dashed lines correspond to the larger and smaller radii respectively). Note that due to different empirical means and different numbers of points in the persistence diagrams, these lines are not the same for the two diagrams, but are very close. The Figures in the middle show the cycles with the largest ℓ-value for $R = 0.4$ (left, significant) and $R = 0.2$ (right, non-significant).

In the next experiment, we used a "cut" annulus, i.e., we take an annulus with a strip removed from it (see Figure 26). The resulting space is contractible, and in particular has no signal to be detected. However, the point-cloud generated on the cut-annulus is very similar to that generated on a complete annulus. Indeed, the persistence diagram for points on the cut-annulus tend to have a single 1-cycle with a long lifetime (see Figure 26, left). Our goal is to show that, using our p-value computation, we can determine that despite its long lifetime, this cycle should not be detected as signal. In Figure 26 (middle left and middle right) we present the largest cycles for two different cut-widths. While these cycles look quite similar, the one on the right is not considered statistically significant, due to its later birth time. In Figure 26(right), we show the average number of signal cycles detected in the cut-annulus as a function of the width of the cut. When the width is very small (as in the middle-left figure), it is impossible to distinguish between the annulus and cut-annulus with just 1,000 points. Indeed, in these cases, we often we falsely detect a signal. However, already for relatively small values (around $W = 0.05$) there are cases where we manage to reject the "fake" signal, and at around $W = 0.15$ we do so perfectly. In other words, using our p-values we can reject cycles, even with relatively long lifetimes, that would otherwise be falsely detected as signal.

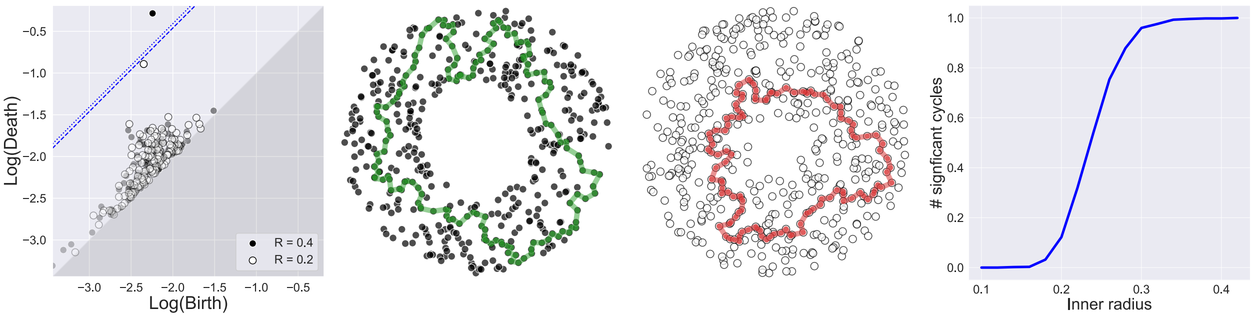

FIGURE 25. Computing p-values for an annulus. (left) Persistence diagrams for two instances with different inner radii, with the significance lines shown, for $\alpha = 0.05$. The dotted line shows the significance threshold for the diagram coming from the larger inner radius, and the dashed line is the threshold for the smaller radius. (middle-left) A significant cycle (p-value $= 0.008$) is detected for $R = 0.4$. (middle-right) The most persistent cycle for $R = 0.2$ is not detected as signal (p-value $= 0.076$), due to the small inner radius. (right) The p-value curve as a function of the inner radius.

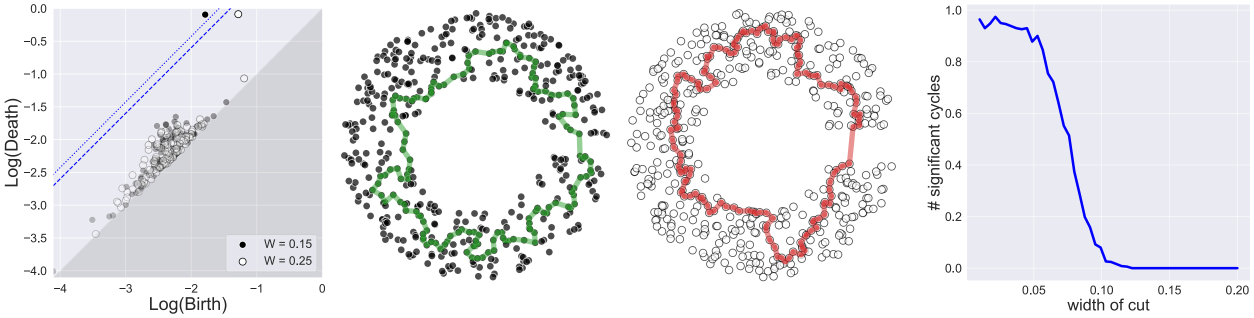

FIGURE 26. Computing p-values for cut-annulus. (left) Persistence diagrams for two instances with different cut-widths, with the significance lines shown for $\alpha = 0.05$ (the dotted and dashed lines correspond to the smaller and larger cuts respectively). (middle-left) At cut-width $W = 0.15$ it is difficult to distinguish the cut-annulus from a complete one, and the cycle is detected as signal (p-value $= 0.008$). (middle-right) A non-significant but persistent cycle (p-value $= 0.16$), with a cut-width of $W = 0.25$. The lack of significance is due to the later birth time of the cycle. (right) The p-value curve for a cut-annulus with a function of the cut-width.

7. Infinite cycles

To demonstrate the power of Algorithm 1, we generated a point-cloud on the 2-dimensional torus, embedded in $\mathbb{R}^3$. The torus radii are $R_1 = 1$ and $R_2 = 2$. Originally, if we wanted to inspect the lifetime of the signal cycles in this example, and possibly to compute a p-value, we need to take the filtration all the way up to $r = R_2$. This will result in a large amount of simplices that would require a massive amount of memory and CPU time. Instead, we ran Algorithm 1, which incrementally increases the filtration threshold just enough to be able to determine significance for all the cycles in the filtration. Table 6 provides some insight into how much computational resources we can save by using this method. For varying sample sizes, we ran this procedure and found the largest value of τ needed to classify all cycles as signal or noise. We compare the number of edges in the Rips complex at radius τ with the number of edges at radius 2 (which, without Algorithm 1, is the only way to compute a p-value for both signal cycles). We observe that the amount of edges we are saving is quite large, and is increasing with the sample size. The saving we expect to

# of points	τ	# of edges at $r = 2$ (1000s)	# of edges at $r = \tau$ (1000s)	ratio
2000	1.98	490	475	1.03
5000	1.67	3067	1876	1.64
10000	1.41	12221	5022	2.43
20000	1.19	49074	13942	3.52
50000	0.95	306580	53581	5.72

Table 6. The number of edges (in 1000s) at the full threshold ($r = 2$) versus the computed threshold τ versus the number of points. The last column represents the ratio of the number of edges.

achieve in the number of triangles is much higher, however, we were unable to compute the exact numbers due to computational limitations.

References

[1] Aaron Abrams and Robert Ghrist. Finding topology in a factory: configuration spaces. *The American mathematical monthly*, 109(2):140–150, 2002.

[2] Henry Adams, Tegan Emerson, Michael Kirby, Rachel Neville, Chris Peterson, Patrick Shipman, Sofya Chepushtanova, Eric Hanson, Francis Motta, and Lori Ziegelmeier. Persistence images: A stable vector representation of persistent homology. *Journal of Machine Learning Research*, 18, 2017.

[3] Robert J. Adler, Sarit Agami, and Pratyush Pranav. Modeling and replicating statistical topology and evidence for CMB nonhomogeneity. *Proceedings of the National Academy of Sciences*, 114(45):11878–11883, November 2017.

[4] Jens Agerberg, Wojciech Chacholski, and Ryan Ramanujam. Data, geometry and homology. *arXiv preprint arXiv:2203.08306*, 2022.

[5] Jean-Baptiste Bardin, Gard Spreemann, and Kathryn Hess. Topological exploration of artificial neuronal network dynamics. *Network Neuroscience*, 3(3):725–743, 2019.

[6] Andrew J. Blumberg, Itamar Gal, Michael A. Mandell, and Matthew Pancia. Robust Statistics, Hypothesis Testing, and Confidence Intervals for Persistent Homology on Metric Measure Spaces. *Foundations of Computational Mathematics*, pages 1–45, 2013.

[7] Omer Bobrowski and Robert J. Adler. Distance functions, critical points, and the topology of random čech complexes. *Homology, Homotopy and Applications*, 16(2):311–344, 2014.

[8] Omer Bobrowski, Matthew Kahle, and Primoz Skraba. Maximally persistent cycles in random geometric complexes. *The Annals of Applied Probability*, 27(4):2032–2060, 2017.

[9] Omer Bobrowski, Sayan Mukherjee, and Jonathan E. Taylor. Topological consistency via kernel estimation. *Bernoulli*, 23(1):288–328, February 2017.

[10] Omer Bobrowski and Primoz Skraba. Homological percolation and the Euler characteristic. *Physical Review E*, 101(3):032304, March 2020.

[11] Omer Bobrowski and Primoz Skraba. Homological percolation: The formation of giant k-cycles. *International Mathematics Research Notices*, 2022(8):6186–6213, 2022.

[12] Karol Borsuk. On the imbedding of systems of compacta in simplicial complexes. *Fundamenta Mathematicae*, 35(1):217–234, 1948.

[13] Peter Bubenik. Statistical topological data analysis using persistence landscapes. *Journal of Machine Learning Research*, 16:77–102, 2015.

[14] Mathieu Carriere, Marco Cuturi, and Steve Oudot. Sliced wasserstein kernel for persistence diagrams. In *International conference on machine learning*, pages 664–673. PMLR, 2017.

[15] Joseph Minhow Chan, Gunnar Carlsson, and Raul Rabadan. Topology of viral evolution. *Proceedings of the National Academy of Sciences*, 110(46):18566–18571, 2013.

[16] Frédéric Chazal, David Cohen-Steiner, and André Lieutier. A sampling theory for compact sets in Euclidean space. *Discrete & Computational Geometry*, 41(3):461–479, 2009.

[17] Frédéric Chazal, David Cohen-Steiner, and Quentin Mérigot. Geometric inference for probability measures. *Foundations of Computational Mathematics*, 11(6):733–751, 2011.

[18] Frédéric Chazal, Brittany Fasy, Fabrizio Lecci, Bertrand Michel, Alessandro Rinaldo, Alessandro Rinaldo, and Larry Wasserman. Robust topological inference: Distance to a measure and kernel distance. *The Journal of Machine Learning Research*, 18(1):5845–5884, 2017.

[19] Frédéric Chazal, Marc Glisse, Catherine Labruère, and Bertrand Michel. Convergence rates for persistence diagram estimation in topological data analysis. *Journal of Machine Learning Research*, 16:3603–3635, 2015.

[20] Frédéric Chazal and André Lieutier. Weak feature size and persistent homology: computing homology of solids in R n from noisy data samples. In *Proceedings of the twenty-first annual symposium on Computational geometry*, pages 255–262. ACM, 2005.

[21] Maria-Veronica Ciocanel, Riley Juenemann, Adriana T Dawes, and Scott A McKinley. Topological data analysis approaches to uncovering the timing of ring structure onset in filamentous networks. *Bulletin of Mathematical Biology*, 83:1–25, 2021.

[22] David Cohen-Steiner, Herbert Edelsbrunner, and John Harer. Stability of persistence diagrams. *Discrete & Computational Geometry*, 37(1):103–120, 2007.

[23] René Corbet, Michael Kerber, Michael Lesnick, and Georg Osang. Computing the multicover bifiltration. *arXiv preprint arXiv:2103.07823*, 2021.

[24] Vin De Silva and Gunnar E. Carlsson. Topological estimation using witness complexes. *SPBG*, 4:157–166, 2004.

[25] Vincent Divol and Frédéric Chazal. The density of expected persistence diagrams and its kernel based estimation. *Journal of Computational Geometry*, 10(2):127–153, 2019.

[26] Brittany Terese Fasy, Fabrizio Lecci, Alessandro Rinaldo, Larry Wasserman, Sivaraman Balakrishnan, and Aarti Singh. Confidence sets for persistence diagrams. *The Annals of Statistics*, 42(6):2301–2339, 2014.

[27] Richard J Gardner, Erik Hermansen, Marius Pachitariu, Yoram Burak, Nils A Baas, Benjamin A Dunn, May-Britt Moser, and Edvard I Moser. Toroidal topology of population activity in grid cells. *Nature*, 602(7895):123–128, 2022.

[28] Jean Ginibre. Statistical ensembles of complex, quaternion, and real matrices. *Journal of Mathematical Physics*, 6(3):440–449, 1965.

[29] Chad Giusti, Eva Pastalkova, Carina Curto, and Vladimir Itskov. Clique topology reveals intrinsic geometric structure in neural correlations. *Proceedings of the National Academy of Sciences*, 112(44):13455–13460, 2015.

[30] Jean-Claude Hausmann et al. On the vietoris-rips complexes and a cohomology theory for metric spaces. *Annals of Mathematics Studies*, 138:175–188, 1995.

[31] AL Herring, Vanessa Robins, and AP Sheppard. Topological persistence for relating microstructure and capillary fluid trapping in sandstones. *Water Resources Research*, 55(1):555–573, 2019.

[32] Yasuaki Hiraoka, Tomoyuki Shirai, and Khanh Duy Trinh. Limit theorems for persistence diagrams. *The Annals of Applied Probability*, 28(5):2740–2780, 2018.

[33] Matthew Kahle and Elizabeth Meckes. Limit the theorems for Betti numbers of random simplicial complexes. *Homology, Homotopy and Applications*, 15(1):343–374, 2013.

[34] Miroslav Kramár, Arnaud Goullet, Lou Kondic, and Konstantin Mischaikow. Persistence of force networks in compressed granular media. *Physical Review E*, 87(4):042207, 2013.

[35] Miroslav Kramár, Rachel Levanger, Jeffrey Tithof, Balachandra Suri, Mu Xu, Mark Paul, Michael F Schatz, and Konstantin Mischaikow. Analysis of kolmogorov flow and rayleigh–bénard convection using persistent homology. *Physica D: Nonlinear Phenomena*, 334:82–98, 2016.

[36] Yongjin Lee, Senja D Barthel, Pawel Dlotko, S Mohamad Moosavi, Kathryn Hess, and Berend Smit. Quantifying similarity of pore-geometry in nanoporous materials. *Nature communications*, 8(1):1–8, 2017.

[37] Melissa R McGuirl, Alexandria Volkening, and Björn Sandstede. Topological data analysis of zebrafish patterns. *Proceedings of the National Academy of Sciences*, 117(10):5113–5124, 2020.

[38] Anthea Monod, Sara Kalisnik, Juan Ángel Patino-Galindo, and Lorin Crawford. Tropical sufficient statistics for persistent homology. *SIAM Journal on Applied Algebra and Geometry*, 3(2):337–371, 2019.

[39] Partha Niyogi, Stephen Smale, and Shmuel Weinberger. A topological view of unsupervised learning from noisy data. *SIAM Journal on Computing*, 40(3):646–663, 2011.

[40] Yohei Onodera, Shinji Kohara, Shuta Tahara, Atsunobu Masuno, Hiroyuki Inoue, Motoki Shiga, Akihiko Hirata, Koichi Tsuchiya, Yasuaki Hiraoka, Ippei Obayashi, et al. Understanding diffraction patterns of glassy, liquid and amorphous materials via persistent homology analyses. *Journal of the Ceramic Society of Japan*, 127(12):853–863, 2019.

[41] Takashi Owada and Robert J. Adler. Limit theorems for point processes under geometric constraints (and topological crackle). *The Annals of Probability*, 45(3):2004–2055, 2017.

[42] Takashi Owada and Omer Bobrowski. Convergence of persistence diagrams for topological crackle. *Bernoulli*, 26(3):2275–2310, 2020.

[43] Theodore Papamarkou, Farzana Nasrin, Austin Lawson, Na Gong, Orlando Rios, and Vasileios Maroulas. A random persistence diagram generator. *Statistics and Computing*, 32(5):88, 2022.

[44] Giovanni Petri, Paul Expert, Federico Turkheimer, Robin Carhart-Harris, David Nutt, Peter J. Hellyer, and Francesco Vaccarino. Homological scaffolds of brain functional networks. *Journal of The Royal Society Interface*, 11(101):20140873, 2014.

[45] Pratyush Pranav, Herbert Edelsbrunner, Rien van de Weygaert, Gert Vegter, Michael Kerber, Bernard J. T. Jones, and Mathijs Wintraecken. The Topology of the Cosmic Web in Terms of Persistent Betti Numbers. *Monthly Notices of the Royal Astronomical Society*, page stw2862, November 2016.

[46] Jan Reininghaus, Stefan Huber, Ulrich Bauer, and Roland Kwitt. A stable multi-scale kernel for topological machine learning. In *Proceedings of the IEEE conference on computer vision and pattern recognition*, pages 4741–4748, 2015.

[47] Donald R Sheehy. A multicover nerve for geometric inference. In *CCCG*, pages 309–314, 2012.

[48] Tomoyuki Shirai and Kiyotaka Suzaki. A limit theorem for persistence diagrams of random filtered complexes built over marked point processes. *arXiv preprint arXiv:2103.08868*, 2021.

[49] Primoz Skraba, Maks Ovsjanikov, Frederic Chazal, and Leonidas Guibas. Persistence-based segmentation of deformable shapes. In *Computer Vision and Pattern Recognition Workshops (CVPRW), 2010 IEEE Computer Society Conference on*, pages 45–52. IEEE, 2010.

[50] Elchanan Solomon, Alexander Wagner, and Paul Bendich. From geometry to topology: Inverse theorems for distributed persistence. *arXiv preprint arXiv:2101.12288*, 2021.

[51] Gábor J Székely, Maria L Rizzo, and Nail K Bakirov. Measuring and testing dependence by correlation of distances. *The annals of statistics*, 35(6):2769–2794, 2007.

[52] Sarah Tymochko, Elizabeth Munch, Jason Dunion, Kristen Corbosiero, and Ryan Torn. Using persistent homology to quantify a diurnal cycle in hurricanes. *Pattern Recognition Letters*, 133:137–143, 2020.

[53] Jasna Urbancic and Primoz Skraba. Patterns emerging from flow. in preparation.

[54] Rien Van De Weygaert, Gert Vegter, Herbert Edelsbrunner, Bernard JT Jones, Pratyush Pranav, Changbom Park, Wojciech A. Hellwing, Bob Eldering, Nico Kruithof, and E. G. P. Bos. Alpha, betti and the megaparsec universe: on the topology of the cosmic web. In *Trans. on Comp. Science*, pages 60–101, 2011.

[55] Lu Xian, Henry Adams, Chad M. Topaz, and Lori Ziegelmeier. Capturing dynamics of time-varying data via topology. *Foundations of Data Science*, 4(1):1–36, March 2022. Publisher: Foundations of Data Science.

[56] D. Yogeshwaran and Robert J. Adler. On the topology of random complexes built over stationary point processes. *The Annals of Applied Probability*, 25(6):3338–3380, 2015.

[57] D. Yogeshwaran, Eliran Subag, and Robert J. Adler. Random geometric complexes in the thermodynamic regime. *Probability Theory and Related Fields*, pages 1–36, 2016.

[1]Faculty of Electrical and Computer Engineering, Technion - Israel Institute of Technology
Email address: omer@ee.technion.ac.il

[2]School of Mathematical Sciences, Queen Mary University of London, UK
Email address: p.skraba@qmul.ac.uk
